# Perspective for Modulation of Hypothalamic Neurogenesis: Integrating Anatomical Insights with Exercise and Dietary Interventions

**DOI:** 10.3390/ijms262210914

**Published:** 2025-11-11

**Authors:** Javier Choquet de Isla, Manuel Bández-Ruiz, Ignacio Rosety-Rodríguez, Inmaculada Pérez-López, Miguel Ángel Rosety-Rodríguez, Cristina Verástegui-Escolano, Ismael Sánchez-Gomar, Noelia Geribaldi-Doldán

**Affiliations:** 1 Departamento de Anatomía y Embriología Humana, Facultad de Medicina, Universidad de Cádiz, 11003 Cadiz, Spaininma.perezlopez@gm.uca.es (I.P.-L.); cristina.verastegui@gm.uca.es (C.V.-E.); 2 INiBICA, Hospital Universitario Puerta del Mar Universidad de Cádiz, Avda. Ana de Viya 21, 11009 Cadiz, Spainmiguelangel.rosety@gm.uca.es (M.Á.R.-R.); ismael.sanchez@gm.uca.es (I.S.-G.); 3 Departamento de Biomedicina, Biotecnología y Salud Pública, Área de Bioquímica, Facultad de Medicina, Universidad de Cádiz, 11003 Cadiz, Spain; 4 Departamento de Didáctica de la Educación Física, Plástica y Musical, Área Educación Física y Deportiva, Facultad de Educación, Universidad de Cádiz, 11519 Puerto Real, Spain; 5 Departamento de Biomedicina, Biotecnología y Salud Pública, Área de Fisiología, Facultad de Medicina. Universidad de Cádiz, 11003 Cadiz, Spain

**Keywords:** hypothalamus, neurogenesis, anatomy, nutraceuticals, metabolism, neural stem cells, tanycytes

## Abstract

Adult neurogenesis is well established in canonical niches—the dentate gyrus and the subventricular zone, where aerobic exercise reliably enhances progenitor proliferation, survival, and synaptic integration via increased cerebral blood flow, neurotrophins (e.g., BDNF, IGF-1), neurotransmitter regulation, and reduced neuroinflammation. Nutraceuticals (e.g., polyphenols, omega-3, creatine, vitamins) further support neuroplasticity and neuronal survival through convergent trophic, anti-inflammatory, and metabolic pathways. By contrast, the hypothalamus, a metabolically pivotal, non-canonical niche, remains comparatively understudied. Here, we synthesize anatomical and functional features of hypothalamic neural stem cells, primarily tanycytes (α1, α2, β1, β2), which line the third ventricle and differentially contribute to neuronal activity regulation, metabolic signaling, and cerebrospinal fluid–portal vasculature coupling, thereby linking neurogenesis to endocrine control. Notably, tanycytes can form neurospheres in vitro, enabling mechanistic interrogation. Although evidence for adult hypothalamic neurogenesis in humans is debated due to methodological constraints, animal data suggest potential relevance to disorders characterized by neuronal loss, metabolic dysregulation, and impaired neuroendocrine function. We propose that an integrative framework is timely: exercise and diet likely interact in the hypothalamic niche through shared mediators (BDNF, IGF-1, CNTF, GPR40) and exercise-derived signals (e.g., lactate, IL-6) that may be complemented by defined nutraceuticals. Yet critical uncertainties persist, including the extent of bona fide hypothalamic neurogenesis, nucleus-specific responses (arcuate nucleus, paraventricular nucleus, ventromedial hypothalamic nucleus), and the mechanistic integration of lifestyle signals in this region. To address these gaps, we outline actionable priorities: (i) single-cell and lineage-tracing studies of tanycyte subtypes under distinct training modalities (aerobic, high-intensity interval training, resistance); (ii) combinatorial interventions pairing structured exercise with nutraceuticals to test synergy on progenitor dynamics and inflammation; and (iii) multi-omics and translational studies to identify biomarkers and establish clinical relevance. Clarifying these interactions will determine whether lifestyle and supplementation strategies can synergistically modulate hypothalamic neurogenesis and inform therapies for neurological, neuropsychiatric, and metabolic disorders.

## 1. Introduction

**Figure 1:**
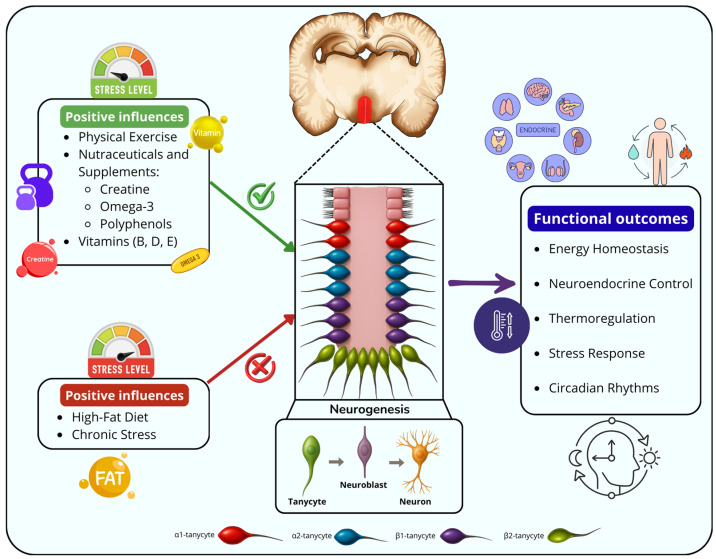
Role of the hypothalamus as a non-canonical niche for adult neurogenesis. Positive modulators (e.g., exercise, nutraceuticals and vitamins) and negative modulators (e.g., a high-fat diet and chronic stress) influence tanycyte-derived neurogenesis. This, in turn, impacts key functional outcomes such as energy homeostasis, neuroendocrine control, thermoregulation, the stress response and circadian rhythms.

Recent studies have increasingly highlighted the existence of adult neurogenesis in both animal models and humans, although not without some controversy, emphasizing its potential as a promising strategy for the treatment and prevention of diseases associated with neuronal loss [1,2,3]. In mammals, neurogenesis is the process by which new functional neurons are generated from neural stem cells (NSCs). Although neurogenesis has been investigated in multiple brain regions, not all areas show the capacity to harbor NSCs [4]. Regions capable of providing a microenvironment that allows the accommodation and differentiation of these cells are known as neurogenic niches [4,5]. The subventricular zone (SVZ) and the subgranular zone of the dentate gyrus (DG) are the two neurogenic regions that are best characterized in the adult mouse brain. In the SVZ, newly generated neuroblasts migrate long distances via the rostral migratory stream to integrate into the olfactory bulb (OB). By contrast, neuroblasts that originate in the DG remain within the hippocampus, where they mature and integrate into the hippocampal circuitry [6,7,8]. Nowadays, the hypothalamus has been identified as an additional neurogenic region [9,10,11]. Given its crucial role in the regulation of metabolic and homeostatic processes, this area has attracted increasing attention from researchers seeking to explore its neurogenic potential [1]. Hypothalamic neurogenesis is a topic that deserves focused attention today, as it represents a paradigm shift in our understanding of adult brain plasticity. Unlike classical neurogenic niches, the hypothalamus integrates systemic metabolic, hormonal and inflammatory cues, thereby linking peripheral physiological states with remodeling of the central nervous system. The main controversy surrounding this potential neurogenic area is the relatively low rate of cell generation compared to canonical niches. However, activating trophic signaling (stimulated by factors such as nutraceuticals or physical exercise) can increase the rate of proliferation due to the presence of specific receptors in this region, which are highly susceptible to systemic and environmental changes [12]. Indeed, the neurogenic potential of the hypothalamus is closely linked to its privileged anatomical location. Positioned at the convergence of multiple neural pathways, the hypothalamus lies at the core of the limbic system, rendering it ideally situated to integrate diverse signals. This strategic location enables the hypothalamus to efficiently detect changes in circulating metabolites and hormone levels, thereby regulating a wide range of physiological processes and behaviors. In adult humans, the hypothalamus is recognized as a central neuroendocrine hub whose primary functions include maintaining energy and fluid homeostasis, thermoregulation, sleep–wake cycles, stress responses, growth and reproductive behaviors. Additionally, the hypothalamus contributes to the regulation of emotional and social behaviors, though these functions are comparatively under-explored in current research [13,14,15]. As previously mentioned, adult neurogenesis represents a key mechanism by which the hypothalamus maintains neuroplasticity, enabling continuous adaptation to dynamic changes in both the internal and external environments. Most studies on postnatal hypothalamic neurogenesis have been centered on its function in the regulation of metabolism and body weight. Several reports have also shown that environmental stimuli such as changes in diet, hormone levels, behavior or exercise can affect hypothalamic neurogenesis [1,16]. As we will discuss in this review, the hypothalamus comprises a specific structure organized into three distinct regions: an ependymal, an internal and an external region. The ependymal region is characterized by its potential neurogenic capacity and its direct contact with the cerebrospinal fluid (CSF) surrounding the third ventricle [17,18,19]. Within this neurogenic region resides a population of cells known as tanycytes [20,21], whose characteristics and functional capabilities will be explored in depth throughout this manuscript. Regarding the ability to stimulate various brain processes, nutraceutical and physical activity has recently become a central focus of attention. The enhancement of neurogenic capacity by various factors, including physical exercise, was discovered many years ago in experimental animal models [22]. Today, in the context of general health, research is increasingly focused on identifying factors that can positively influence overall well-being. In this regard, and as part of a healthy lifestyle, which includes regular physical activity, scientists are actively investigating a number of agents that can contribute to brain and systemic health. Among these approaches, nutraceuticals have emerged as compelling candidates. These food-derived bioactive compounds offer physiological benefits that extend beyond simple nutritional support [23,24]. Thus, referring to the supplements that are used for balanced diets, they consider minerals and vitamins among other issues that complement the diet by increasing its total intake [25]. Several nutraceuticals have been registered that have effects on various pathologies ranging from Alzheimer’s disease [26,27], aging [28,29,30], cardiovascular diseases [31,32], cancer [33,34,35], diabetes [33,36] and others. Particularly, today, there are numerous sports supplements, which are nutraceuticals, and which include specific compounds with a potential effect on various processes, such as creatine, which is positioned as one of the star supplements with effects on various mechanisms, including the improvement of neurogenesis in areas such as the hippocampus, and which are evidenced to have broad benefits at the cognitive level [37,38]. Altogether, exercise and diet act through convergent molecular pathways. These influence neurogenesis, inflammation, energy metabolism, and synaptic plasticity. They both modulate overlapping signaling cascades, including BDNF/TrkB, AMP-activated protein kinase (AMPK) and SIRT1, which ultimately affect neuronal survival and plasticity [39,40]. Their combined analysis is particularly relevant in the hypothalamic niche, which is a key interface between peripheral metabolic cues and central adaptability. There is evidence to suggest that exercise-induced trophic signaling may enhance the neurogenic and anti-inflammatory effects of nutraceuticals, thereby supporting a synergistic impact on cerebral plasticity [41]. However, current therapeutic approaches rarely address these integrative mechanisms. The aim of this review is therefore to summarize the emerging evidence linking exercise and nutraceuticals in the modulation of hypothalamic neurogenesis, and to highlight their potential translational relevance for metabolic and neuropsychiatric disorders. In this integrative framework, examining pathophysiological scenarios in which diet and exercise influence processes such as neuroregeneration becomes particularly relevant. For instance, regular exercise and nutraceuticals protect the nervous system through complementary mechanisms. Exercise modulates redox balance by producing transient reactive oxygen species, which activates antioxidant defenses, promotes NSC proliferation and regulates BDNF expression [42]. In turn, nutraceuticals target shared pathological pathways involved in neurodegeneration, such as mitochondrial dysfunction, calcium imbalance, oxidative stress and inflammation, through multifactorial physiological actions with minimal adverse effects [43]. Together, these lifestyle-based interventions can prevent neuronal damage and attenuate the progression of disorders such as Alzheimer’s disease, Parkinson’s disease, Huntington’s disease, multiple sclerosis and amyotrophic lateral sclerosis [44,45,46]. On the other hand, the combined effects of exercise and diet on neurogenesis have been observed in other, more well-established neurogenic niches. However, there is still limited evidence regarding hypothalamic neurogenesis and how an integrative approach could address conditions associated with neuronal loss [47]. The significance of our review becomes evident in emphasizing the need to examine related mechanisms of exercise and diet in an integrated manner within a central region such as the hypothalamus. Although the hypothalamus is traditionally considered a non-canonical and lesser-explored neurogenic niche, it plays a pivotal role in maintaining systemic homeostasis by integrating metabolic, endocrine and behavioral signals. Understanding how lifestyle factors such as physical activity and nutritional interventions influence hypothalamic plasticity could reveal novel pathways for neural regeneration and metabolic regulation. Our aim is therefore to highlight the hypothalamus as a promising yet underappreciated target for experimental research, encouraging future studies to address its neurogenic capacity from cellular and systemic perspectives. This is illustrated in Figure 1.

## 2. Materials and Methods

The present narrative review aims to integrate various anatomical aspects related to hypothalamic neurogenesis with different elements concerning nutraceuticals, metabolic pathways, and physical exercise. We recognize that research in this non-canonical niche remains limited and that this topic is underdeveloped. Nevertheless, this is an area that has gained particular relevance in recent years. We conducted a literature review based on a keyword search using PubMed, Scopus and Web of Science. The search covered publications from 2001 to 2025, with a particular focus on 2020–2025. However, it should be noted that some earlier studies have been included solely because they are relevant to the topic addressed and provide essential information for this work. The analysis included articles that met the following criteria: English language publications in peer-reviewed scientific journals. Particular attention was given to the impact and quality of the sources, with priority given to articles published in the Q1 and Q2 journals. The exclusion criteria applied to studies included conference abstracts, editorials and letters to the editor, as well as studies for which the full text was unavailable. The search strategy employed the Boolean operators AND and OR to combine the following terms and their corresponding MeSH headings, ensuring comprehensive coverage of the topic. The following search strategy was used: (“Neurogenesis” [MeSH] OR “Neural Stem Cells” [MeSH]) AND (“Hypothalamus” [MeSH] OR “Tanycytes” [All Fields]) AND (“Exercise” [MeSH] OR “Physical Activity” [All Fields]) AND (“Dietary Supplements” [MeSH] OR “Nutraceuticals” [All Fields] OR “Functional Foods” [MeSH]). AND (“Brain Plasticity” [MeSH] OR “Energy Metabolism” [MeSH]).

## 3. Results

### 3.1. Anatomy of the Hypothalamus and the Third Ventricle

The hypothalamus is a key region that integrates sensory information and regulates essential physiological processes. Composed of several nuclei, it controls hunger, thirst, the sleep–wake cycle, and body temperature. The hypothalamus modulates the endocrine system through its connections with the pituitary gland. The hypothalamus interacts with the limbic system to regulate emotions and social behavior [48,49]. The hypothalamus is connected to various brain structures, including the reticular formation, thalamus, amygdala, hippocampus, OB, retina, and cerebral cortex [50]. This allows the hypothalamus to influence multiple everyday activities [51]. The hypothalamus is part of the diencephalon. It is located beneath the thalamus and is separated from it by the hypothalamic sulcus of Monro. This sulcus is situated in the lateral wall of the third ventricle. Anteriorly, the hypothalamus is bounded by the anterior commissure and the lamina terminalis, which extends above the optic chiasm between the two anterior columns of the fornix. The lamina terminalis constitutes the anterior wall of the third ventricle and contains the vascular organ of the lamina terminalis (OVLT). This structure is characterized by the absence of a blood–brain barrier. Consequently, the OVLT is highly sensitive to osmotic variations in the blood. Its neurons can detect extracellular concentrations of NaCl and angiotensin II, which contributes to the regulation of body fluid homeostasis and blood pressure [52]. The superior boundary of the hypothalamus constitutes part of the inferolateral wall of the third ventricle. In this region, it is located next to the fornix, which is a C-shaped pathway made up of white matter that connects the hypothalamic nuclei to the hippocampus and the thalamic nuclei to the mammillary bodies. The mammillary bodies are small, rounded white matter structures that are part of the limbic system. Due to their connections with the hippocampal formation, they are involved in memory processing and play a role in maintaining spatial orientation. Dorsally, the hypothalamus extends toward the periaqueductal gray matter and the tegmentum of the upper brainstem. Anteriorly, the hypothalamus is related to the optic chiasm and the anterior perforated substance; posteriorly, it is related to the cerebral peduncles of the midbrain and the mammillary bodies. In this region, the tuber cinereum, a gray matter structure, projects downward to form the infundibulum, also known as the pituitary stalk. The pituitary stalk connects the hypothalamus to the posterior lobe of the pituitary gland, or neurohypophysis, which is located within a small depression of the sphenoid bone known as the sella turcica [1,15,17]. The human hypothalamus has three main anteroposterior regions: the supraoptic region, the tuberal region, and the mammillary region. The anterior region of the hypothalamus, also known as the supraoptic area, contains several nuclei, including the supraoptic (SON), preoptic, medial preoptic (MPO), suprachiasmatic (SCN), and anterior hypothalamic nuclei (AHN). The SON produces vasopressin, also known as the antidiuretic hormone (ADH), which is stored in the posterior lobe of the pituitary gland. Vasopressin plays a crucial role in regulating blood pressure and body fluid balance [53]. The preoptic region and the AHN are involved in thermoregulation. The preoptic nucleus regulates appetite and reproductive functions. The MPO controls the cardiovascular response to stress [54,55]. The SCN, located above the optic chiasm, is involved in regulating the circadian rhythm. The tuberal region consists of anterior and lateral parts that contain the dorsomedial hypothalamic nucleus (DMH), the ventromedial hypothalamic nucleus (VMH), the paraventricular nucleus (PVN), and the arcuate nucleus (ARC) (or infundibular). The VMH plays a key role in controlling appetite and the sensation of satiety [56,57]. The PVN, a major autonomic center in the brain, is involved in regulating stress responses and metabolism [58]. The ARC secretes orexigenic peptides, including ghrelin, orexin, and neuropeptide Y (NPY) [59]. The posterior hypothalamic nucleus and the mammillary nucleus are located in the posterior or mammillary region. Although they are anatomically part of the hypothalamus, they functionally belong to the limbic system. The entire hypothalamic region is associated with energy balance, blood pressure regulation, memory, and learning due to its connections with the hippocampus and Papez circuit [60]. An overview of the hypothalamus, its different nuclei, and the signaling pathways associated with each of these nuclei is provided in Table 1. The unique anatomical organization of the hypothalamus, particularly its proximity to the third ventricle and the presence of NSCs lining its walls, provides the structural basis for hypothalamic neurogenesis, positioning these cells as key mediators between the CSF, vasculature, and neural plasticity [18,61]. In the same way, this configuration makes the hypothalamus especially susceptible to metabolic changes that can be modulated and improved through diet and physical exercise, as will be further analyzed in the following sections.

### 3.2. Hypothalamic Neurogenic Niche: The Tanycytes as Hypothalamic Stem Cells

In order to provide context for the subject addressed in this review, it is important to discuss adult neurogenesis and its implications. In general, adult neurogenesis is a significant event related to brain plasticity and memory formation. The SVZ and the DG of the hippocampus are the most extensively studied regions to date [114,115,116]. Despite the ongoing controversies surrounding its existence and functional relevance, this phenomenon is highly conserved across species, from rodents to primates, including humans [6,117,118,119], and is now a key focus in the study of neuronal regeneration. Consistent with the above, the discovery of neurogenesis in the hypothalamus is a topic that remains controversial. Despite this, studying this brain region is crucial because of the important functions it performs. During embryonic development, the hypothalamus originates from the rostral diencephalon and becomes regionally specified along the anteroposterior and dorsoventral axes through the spatiotemporal regulation of morphogens and transcription factors [120]. Cell production follows general neurogenic principles: periventricular radial NSCs first generate neurons, followed by glial cells. In this context, hypothalamic NSCs correspond to radial glial cells that come into contact with both the ventricular and pial surfaces, exhibiting interkinetic nuclear migration. These ventricular radial glia give rise to a secondary population of radial glia-like cells that maintain pial contact, but lose their apical process. These cells undergo mitotic somatic translocation before producing neuronal and progenitor cells that migrate to their final destinations [121]. In mice, hypothalamic neurogenesis occurs between E9 and E18, with the majority of neurons being formed between E11 and E14 [121]. However, the onset and progression of astrocyte formation remain unclear. Initially, Sonic Hedgehog (Shh)-expressing progenitors produce both neurons and astrocytes, but after E12.5 they become predominantly gliogenic, showing region-specific contributions across hypothalamic domains. Advances in single-cell transcriptomics have begun to reconstruct developmental hierarchies and regulatory networks. These studies have highlighted the activation of the Notch pathway during the transition from gliogenic progenitors to astrocytes. However, the molecular mechanisms of astrocyte differentiation remain incompletely defined [122]. Due to its immature characteristics and proximity to the third ventricle, it was suggested that these neurogenic capabilities could persist into adulthood, similar to those observed in more extensively studied canonical niches. The complex structure of the hypothalamus confers specific functional capacities because its anatomy and organization are intimately linked to its function. As mentioned earlier, it is part of the diencephalon and aligned with the third ventricle along the midline, which has led to the suggestion that it could be a neurogenic niche. This idea has become increasingly relevant in recent years, establishing the hypothalamus as a focal point of current research. In line with this idea, it is worth highlighting that the median eminence (ME) of the hypothalamus plays a key structural role in regulating neurogenesis in this region. Anatomically, it is divided into three zones: the ependymal, internal, and external zones. The ependymal zone forms part of the floor of the third ventricle and is significant because it contains specialized glial cells called tanycytes. These specialized cells are also present during embryonic development [123]. Indeed, these cells have microvilli that extend into the ventricular cavity and interact with the CSF. They also have long cytoplasmic processes that reach into the ME. This unique morphology enables tanycytes to contribute to the formation of a selective barrier within the brain [13,17,124,125,126]. The first evidence of neurogenesis in this region dates to the 2004 study by Markakis et al., in which neural progenitor cells were successfully isolated from this area for the first time [127]. The aforementioned study identified several peptides, such as corticotropin-releasing hormone (CRH), growth hormone-releasing hormone, gonadotropin-releasing hormone, somatostatin, thyrotropin-releasing hormone, oxytocin and vasopressin, in hypothalamic progenitor cells. This finding proposed the hypothalamus as a novel neurogenic niche in adults and highlighted the expression of peptides and proteins typically associated with mature neurons. Another study demonstrated that these cells were capable of migrating from the walls of the third ventricle into the hypothalamic parenchyma in rats, specifically towards the dorsomedial, anterolateral and ventrolateral regions of the hypothalamus [128]. Subsequent studies have identified neurogenically active regions associated with specific hypothalamic functions [129,130]. Neurogenesis in this region requires specific cytoarchitecture that is closely associated with the third ventricle and specialized cells known as tanycytes [9,20,61]. As mentioned above, tanycytes retain characteristics of radial glia, including their location around the third ventricle, morphology, and function. In terms of gene expression, they share numerous markers with other NSCs, including Sox2, Sox9, nestin, and vimentin [129,131]. There are different types of tanycyte, each of which is located specifically around the third ventricle, specifically located in the so-called hypothalamic proliferative zone, which encompasses the basal portion of the hypothalamus. This includes the ME and the lateral walls at the level of the PVN and ARC nuclei. Although the underlying mechanisms are not yet fully understood, tanycytes exhibit increasing heterogeneity during maturation, which is likely to mirror their functional specialization. Broadly speaking, tanycytes are classified into four major subtypes based on their dorsoventral positioning, α1, α2, β1, and β2 [132,133]. These tanycytes are located in a specific region of the hypothalamus. Considering the tuberal structure surrounding the third ventricle, three regions can be identified: a dorsal region characterized by a lack of proliferative activity; a middle zone with features similar to the typical proliferative region of the SVZ; and a ventral zone associated with the ME related to ependyma tanycytes [134]. Indeed, according to studies by Lee et al., ME has been proposed as the most neurogenic area of the hypothalamus. However, other studies have shown that the intermediate zones, including the periventricular and paraventricular regions, also exhibit a certain degree of proliferative activity [129]. However, this proliferative activity may be associated with specific stimuli, such as certain growth factors [135]. According to studies conducted by Rodríguez and colleagues, α1 tanycytes are typically found in the VMH and DMH nuclei, α2 tanycytes are usually located bordering the ARC, and β1 tanycytes occupy the lateral regions surrounding the third ventricle. β2 tanycytes are found on the floor of the third ventricle [133]. This subdivision has been corroborated by gene expression studies [136] and, more relevantly for the purposes of this review, by the analysis of various neural markers. In this context, one way to distinguish α-tanycytes from β-tanycytes is by their expression of GLAST (Glutamate Aspartate Transporter), which is found exclusively in α-tanycytes. Furthermore, only α-tanycytes have demonstrated the ability to form neurospheres in culture. Interestingly, even within the α-tanycyte population, heterogeneity has been observed; only cells positive for the glial marker GFAP are capable of forming neurospheres. This suggests the existence of functionally distinct subpopulations [10]. Conversely, it has been reported that β-tanycytes located near the ARC exhibit neurogenic potential, as detailed in a study by Hann et al. In this study, the authors explain that this cell type cannot be identified using GLAST or GFAP markers but instead displays an FGF10 (Fibroblast Growth Factor 10) immunoprofile [9]. There is ongoing controversy regarding the different tanycyte subtypes and their respective roles in the neurogenic process in this region. Some studies suggest that α-tanycytes predominantly give rise to glial cells [137]. However, others argue that GFAP-positive α-tanycytes closely resemble NSCs found in the SVZ in terms of both molecular profile and functional potential [10,11]. In summary, evidence supporting the existence of neurogenesis in this area has been very promising in recent years. However, as with other non-canonical niches, much of the research in this area has focused on pathological conditions. For instance, with regard to obesity, researchers have reported that a sustained high-fat diet (HFD) and leptin deficiency impact neurogenesis in the ARC. This was demonstrated in a study by McNay et al., in which the ratio of BrdU-labeled cells over time was analyzed, showing impaired neurogenesis [138]. Other studies related to obesity have been conducted in the context of inflammation. For example, interleukin-6, a molecule produced during physical exercise, has been shown to induce the expression of genes associated with neurogenesis in the hypothalamus [139]. The hypothalamus plays a crucial role in maintaining body temperature homeostasis through various regulatory mechanisms. The preoptic area (POA) acts as the main control center, while other hypothalamic regions influence thermoregulatory behaviors. Chronic heat exposure prompts adult neurogenesis, particularly within the POA, which in turn facilitates heat acclimation. This is characterized by a drop in core body temperature and an improvement in the body’s ability to withstand heat stress [140,141]. In other conditions, such as stroke, it has been demonstrated that voluntary exercise can enhance the recovery of hypothalamic homeostasis by improving energy balance and promoting the proliferation and differentiation of hypothalamic cells [142]. On the other hand, a significant effect on hypothalamic neurogenesis has also been demonstrated in response to stress, resulting in reduced proliferation and differentiation in regions such as the ME and the ARC [143]. Hypothalamic neurogenesis is currently also being implicated in relation to stress and major depressive disorder. Studies have found that depression reduces neurogenesis in the hypothalamus and increases dendritic atrophy. In this context, studies by Solak et al. suggest that modulation through NPY1R agonists could be a key approach to treating this condition [144]. Hypothalamic neurogenesis is currently emerging as a promising avenue for identifying novel therapeutic targets for conditions characterized by neuronal loss. Indeed, numerous studies have sought to elucidate which tanycyte subtypes are involved and what their specific roles are. α1-tanycytes are located in the VMH and the DMH nuclei, where they extend their processes into the brain parenchyma, as we mentioned before. There, they form a barrier and communicate with neurons, contributing to the regulation of energy metabolism and reproduction [133,145,146]. α2-tanycytes, which are found in the dorsomedial Arcuate region, create barriers with blood vessels and neurons and perform functions that are similar to those of α1-tanycytes [146,147]. β1-tanycytes reside in the ventromedial Arcuate nucleus of the Hypothalamus and project to the lateral ME and Arc nucleus. They are nutrient-sensitive cells whose barrier properties vary depending on metabolic status, playing a key role in energy homeostasis [146,148,149]. Finally, β2-tanycytes are located at the floor of the third ventricle and contact the fenestrated capillaries of the ME. Their tight junctions are crucial for maintaining the blood–hypothalamus barrier and regulating the entry of hormones and nutrients into the brain, thereby influencing energy balance and reproductive function [145,147]. However, the specific tanycyte subtypes affected by particular diseases are still under investigation. Although some insights are beginning to emerge, more in-depth studies are required, as mentioned earlier. Unlike other regions of the brain traditionally associated with neurogenesis, such as the hippocampus or the SVZ, the hypothalamus has a distinct neurogenic niche which remains functionally active throughout adulthood. This niche is closely linked to vital physiological processes including energy homeostasis, the stress response and neuroendocrine regulation. Considering these implications, Table 2 provides a detailed breakdown of the various hypothalamic nuclei, emphasizing those for which evidence of neurogenesis has been documented.

The ability to modulate hypothalamic neurogenesis could lead to new approaches for treating various neurological and psychiatric disorders, such as neurodegenerative diseases, metabolic syndromes and mood disorders, where neuronal damage and impaired neural plasticity are key features. Therefore, harnessing this endogenous regenerative potential is an exciting and under-explored strategy for developing targeted therapies.

### 3.3. Metabolic Regulation in the Hypothalamus and Its Relationship with Neurogenesis

The hypothalamus acts as a central regulator of homeostasis, controlling many metabolic and physiological processes. These include appetite, energy expenditure, thermoregulation, stress response, sleep–wake cycles, fluid balance and osmotic homeostasis. In this section, we will explore the relationship between metabolic pathways and neurogenesis in the hypothalamus. Moreover, it plays a pivotal role in the hypothalamic–pituitary axis by acting as a control center that coordinates signals from the nervous system and regulates pituitary activity. Together, these systems establish a core communication and regulatory network for hormonal signaling, which is essential for maintaining the body’s internal balance. In this context, hypothalamic neurogenesis contributes to neuroplasticity and adaptive plasticity, both of which are crucial for enabling the hypothalamus to respond continuously to changes in the internal and external environments [4]. Newly formed cells are capable of integrating into neuronal circuits and synthesizing and releasing hormones, neurotransmitters, and neuropeptides, thereby contributing to hypothalamic function [123,134].

#### 3.3.1. Regulation of Appetite and Energy Expenditure

The hypothalamus is the main regulator of energy balance and instinctive behaviors, including food intake, through a highly integrated system that combines hormonal, neural, and metabolic signals to modulate appetite and energy expenditure. Key signals include circulating metabolites such as glucose, free fatty acids and amino acids, as well as specific hormones such as leptin, ghrelin and insulin. These act on the ARC, the PVN and other hypothalamic regions to coordinate the body’s response to fluctuations in energy availability [21,126]. The ARC contains two distinct neuronal populations: Neuropeptide Y/agouti-related peptide (NPY/AgRP) neurons secrete orexigenic neuropeptides NPY and AgRP, thereby stimulating appetite. Pro-opiomelanocortin/cocaine- and amphetamine-regulated transcript (POMC/CART) neurons, in contrast, secrete anorexigenic neuropeptides POMC and CART, thereby suppressing appetite. Leptin and insulin have been demonstrated to play a pivotal role in the regulation of these neurons. Leptin, secreted by adipose tissue in proportion to fat stores, acts as a satiety modulator by stimulating POMC/CART neurons and suppressing NPY/AgRP neurons, resulting in reduced appetite and increased energy expenditure. In a similar manner, insulin, the secretion of which in the pancreas is increased in response to elevated glucose levels, acts in a synergistic manner with leptin. This results in the activation of POMC/CART neurons and the inhibition of NPY/AgRP neurons, thereby reinforcing the anorexigenic signal [163,164,165,166]. Conversely, ghrelin, an orexigenic hormone primarily secreted by the stomach during fasting, exerts the opposite effect. The action of the hormone on NPY/AgRP neurons has been demonstrated to stimulate food intake and decrease energy expenditure, thereby promoting a state of positive energy balance. In this manner, ghrelin counteracts the effects of leptin and insulin, thereby facilitating the metabolic adaptation of the organism to varying nutritional states [167,168]. In this specific context, tanycytes have been proposed to act as neuromodulatory cells, regulating the availability and transport of peripheral hormones, such as leptin [169] and ghrelin [170], to neurons in the ARC. Furthermore, tanycytes express glucose sensors that enable them to detect changes in energy availability and relay metabolic signals to hypothalamic neurons [171]. Due to their strategic location, they have the capacity to respond to mitogenic and neurodifferentiating signals present in the peripheral blood or CSF. This contributes to neuronal plasticity and metabolic homeostasis [10,137]. With a focus on intracellular metabolic pathways, AMPK and mechanistic target of rapamycin (mTOR) have been identified as central regulators of energy expenditure. AMPK is an energy sensor that becomes activated under conditions of low cellular energy, such as high AMP/ATP or ADP/ATP ratios. This activation promotes appetite and reduces energy expenditure by stimulating the activity of NPY/AgRP neurons [172]. Conversely, mTOR is known to be activated in the presence of nutrients such as amino acids and glucose, thereby stimulating POMC/CART neurons to promote satiety and increase energy expenditure [173]. Taken together, the balance of these hormones, neuropeptides and signaling pathways enables the hypothalamus to efficiently regulate appetite and energy expenditure, thereby adapting to the organism’s metabolic needs. Disruptions to these mechanisms, or dysfunctional hypothalamic neurogenesis, can significantly contribute to the development of metabolic disorders such as obesity and insulin resistance [16,174,175].

#### 3.3.2. Thermoregulatory Control

Regulation of body temperature is essential for survival and relies on precise control by the nervous system. The hypothalamus plays a key role in this process, functioning as a thermostat that detects and adjusts the body’s internal temperature to keep it within an optimal range. It receives input from thermoreceptors, such as TRPM8, TRPV1 and TRPM2, which are distributed throughout the body and are also present in the hypothalamus [176]. These receptors act as temperature sensors, sending signals to the hypothalamus when they detect changes in temperature. In response to a drop in temperature, cold-sensitive receptors such as TRPM8 activate mechanisms that conserve and generate heat. These mechanisms include vasoconstriction and thermogenesis in brown adipose tissue, which is the primary thermogenic organ responsible for maintaining body temperature through heat production. The hypothalamus also influences beige adipose tissue, which develops from white adipose tissue, promoting lipid oxidation and heat generation [177]. In contrast, heat-sensitive receptors such as TRPV1 and TRPM2 trigger responses, including vasodilation and sweating, in response to elevated temperatures, thereby facilitating heat dissipation [178]. The hypothalamus integrates thermal signals and regulates thermogenesis by activating various metabolic pathways. For example, the nutrient- and hormone-sensitive protein complex mTORC1 plays a vital part in promoting heat production in brown and beige adipose tissue. This process is modulated by hormones such as leptin and insulin, which enhance thermogenesis by activating mTORC1 and specific receptors in POMC neurons, as well as other energy-related pathways. Additionally, the sympathetic nervous system, under hypothalamic control, releases norepinephrine. This stimulates the expression of uncoupling proteins, such as UCP1, in adipocytes, thereby promoting the conversion of energy into heat. Thus, the hypothalamus acts as a control center, adjusting the body’s thermal responses to maintain an optimal internal temperature by increasing or decreasing heat production as required [179]. For example, studies conducted by Benevento and colleagues demonstrated that tanycytes are activated in response to an acute thermal stimulus, leading to a reduction in food intake. The researchers proposed that tanycytes may promote the production of vascular endothelial growth factor A, which acts on the ARC [180]. Although several review articles have addressed this topic, further in-depth studies are needed to elucidate the specific relationship between hypothalamic neurogenesis, tanycytes and temperature regulation. Most of the available evidence originates from animal models, particularly focusing on the POA of the hypothalamus, where thermal stimulation has been demonstrated to induce neuronal plasticity via heat acclimation mechanisms [181].

#### 3.3.3. Hydric Homeostasis and Osmoregulation in the Hypothalamus

The hypothalamus plays a crucial role in regulating water and osmotic balance via multiple mechanisms. It detects changes in plasma osmolality via osmoreceptors located in the OVLT and the subfornical organ. These signals are then integrated within the median preoptic nucleus (MnPO). This activation stimulates the thirst center in the lateral hypothalamus, promoting water intake. Additionally, the supraoptic and PVN synthesize vasopressin (the ADH), which is released from the neurohypophysis in response to increased plasma osmolality or decreased blood volume. Vasopressin acts on the kidneys to enhance water reabsorption, thereby reducing urine output. The hypothalamus also regulates the autonomic nervous system by modulating vasoconstriction via sympathetic tone to maintain blood pressure. In parallel, it interacts with the renin–angiotensin–aldosterone system, which is activated during hypovolemia or hypotension. Angiotensin II stimulates the thirst center and vasopressin release, as well as promoting aldosterone secretion. Aldosterone enhances sodium and water retention in the kidneys [182,183]. To date, we have not found any evidence or published studies linking neurogenesis or tanycytes to hydric homeostasis, opening up a novel field of research yet to be explored. One potential area for future research could be to explore how the hypothalamus regulates hydric homeostasis and influences NSC activity and neurogenesis. Changes in osmotic pressure in the CSF or extracellular environment, as well as vasopressin-mediated signaling, may alter the microenvironment of the neurogenic niche, affecting progenitor proliferation and differentiation. Investigating these interactions could shed light on how hydration status and osmotic balance influence hypothalamic plasticity and associated metabolic processes.

#### 3.3.4. Hypothalamic Regulation of Stress, Circadian Rhythms, and Sleep–Wake Cycles

The hypothalamus regulates stress, circadian rhythms and sleep–wake cycles via integrated mechanisms. In response to stress, it activates the hypothalamic–pituitary–adrenal axis by releasing CRH. This stimulates the pituitary gland to secrete adrenocorticotropic hormone (ACTH). ACTH then promotes the release of cortisol from the adrenal glands. This process is tightly regulated by negative feedback: cortisol acts on glucocorticoid receptors (GR) in the hypothalamus and pituitary to inhibit the production of CRH and ACTH, thus preventing an excessive stress response [184]. Indeed, a recent study evaluated effects on hypothalamic regulation and the Hypothalamic–Pituitary–Adrenal axis (HPA), demonstrating that herbal interventions can modulate hypothalamic–pituitary function [185]. Also, in this context, the PVN of the hypothalamus has been identified as a key regulator of metabolism, stress responses and physiological homeostasis. The GR plays a critical role in mediating these diverse PVN functions. A recent study revealed that early-life stress reduces the number of hypothalamic stem cells and impairs cell proliferation in adult mice [186]. As previously mentioned, there is a growing body of evidence showing that stress, whether moderate or severe, has a detrimental effect on neurogenesis [143]. Regarding circadian cycles, the SCN, which is located in the hypothalamus, acts as the body’s master clock. It synchronizes biological rhythms with the light/dark cycle by regulating clock genes such as CLOCK and BMAL1. These genes control molecular and hormonal oscillations, including the nocturnal release of melatonin by the pineal gland [187,188]. Recent studies have shown that BrdU^+^ cells, which are a marker of cell proliferation, have been found in several regions of the hypothalamus that are involved in sleep–wake regulation. This suggests that adult neurogenesis may contribute to the plasticity of the neural circuits that underlie circadian and sleep-related functions (reviewed in [189]). Furthermore, the POA of the hypothalamus plays a pivotal role in regulating sleep [190,191]. During sleep, active neurons located in the ventrolateral POA and the MnPO express the inhibitory neurotransmitters GABA and galanin [192]. These neurons suppress major arousal-promoting systems in the brainstem and hypothalamus. In contrast, the hypothalamic systems that primarily regulate wakefulness include the hypocretin (orexin) and histamine systems, molecules that promote wakefulness and prevent abrupt transitions between sleep and wake states. These systems play essential roles in promoting and stabilizing wakefulness, as well as regulating muscle tone. Sleep, wakefulness, and circadian rhythms are fundamental for adaptation, survival, and optimal performance in dynamic and demanding environments. Therefore, it is essential to preserve the proper functioning of hypothalamic structures and the cells that regulate these processes. Various factors, such as lifestyle, diet, environment and aging, can alter these systems and impair their efficiency. It has been proposed that hypothalamic neurogenesis contributes to hypothalamic plasticity and its ability to adapt to environmental changes. However, its functional impact on the regulation of sleep and circadian rhythms remains under investigation [189].

Further studies are clearly needed to address the specific effects of neurogenesis on various metabolic pathways involving the hypothalamus. However, this line of research is important because it could help elucidate the impact of different pathological conditions and contribute to the discovery of new therapeutic targets aimed at mitigating dysfunctions in these pathways. One promising area for future research could be to investigate how the hypothalamus regulates stress, circadian rhythms and sleep–wake cycles, and how this influences neurogenesis. Stress-related hormones, such as corticosterone, and circadian oscillations in metabolic and hormonal signals may influence the proliferation and differentiation of NSC in the hypothalamic niche. Understanding these interactions could help to explain how disruptions to stress or sleep regulation affect hypothalamic plasticity and contribute to neurodegenerative and mood disorders.

### 3.4. Effects of Physical Exercise on Hypothalamic Neurogenesis

It has been some time since the wide-ranging benefits of physical exercise were first discovered, both under physiological and pathological conditions. This is particularly evident in neurodegenerative diseases, where physical activity has been shown to significantly enhance neuroplasticity and neurogenesis. This helps to mitigate the devastating effects of such disorders [193,194]. In this regard, neurogenic capacity and neuroplasticity depend on the type of exercise performed. Various animal models have been investigated to elucidate the underlying molecular and cellular mechanisms. It is therefore important to distinguish between different types of exercise, such as aerobic training, anaerobic training and resistance training [195,196]. The positive effect of aerobic exercise on the transport and distribution of nutrients and various growth factors has been demonstrated. BDNF has been identified as the key mediator in enhancing memory and cognitive function [197,198]. In recent years, the term ‘exerkine’ has emerged to refer to bioactive molecules induced by physical exercise. These molecules have been identified as the key mediators of the beneficial effects of physical activity [199]. Various types of exerkines have been identified in human tissues, including interleukin (IL)-6, IL-7, IL-15, BDNF, fibroblast growth factor (FGF) 21, and vascular endothelial growth factor, among others. These molecules can act via paracrine, autocrine, or dual mechanisms, thereby contributing to the systemic effects of exercise [200]. With regard to the brain, the hippocampus is undoubtedly the most extensively studied region, where the profound effects of physical exercise on neurogenesis have been clearly demonstrated [201,202,203]. In fact, it has been demonstrated that both anaerobic resistance training and high-intensity interval training (HIIT) can affect hippocampal neurogenesis [204,205]. Clearly, one of the main challenges of this review is to specify the most effective type of exercise for different brain regions and emphasize the various variables that must be considered, such as exercise intensity, volume and frequency. Ultimately, it is necessary to determine the specific training program to be applied [206]. There is currently substantial scientific evidence from studies demonstrating the effects of anaerobic or resistance exercise on neurogenesis, particularly in the hippocampus. In fact, Jiang et al. reported that high-intensity aerobic exercise significantly enhances adult hippocampal neurogenesis compared to controls, whereas low-intensity exercise only produces a modest increase. No significant changes in synaptic plasticity were observed, which suggests that high-intensity aerobic training may be the most effective way to stimulate adult hippocampal neurogenesis [207]. Others, such as Zhao et al., have demonstrated that aerobic exercise can mitigate the pathology associated with Parkinson’s disease and support adult hippocampal neurogenesis by reducing inflammation in microglia. In their 10-week study involving MPTP-treated mice, they found that exercise enhanced neurogenesis and memory performance, while reducing neuronal apoptosis, microglial activation and NLRP3 inflammasome signaling. They identified the irisin/NLRP3 pathway as a key mediator: exercise-induced irisin counteracts α-synuclein-driven inflammation, and blocking irisin signaling diminishes these neuroprotective effects. Overall, these results emphasize the potential of aerobic exercise as a non-pharmacological approach to preserving neurogenesis and cognitive function by modulating neuroinflammatory pathways [208]. Other studies have shown that physical exercise has an impact on other neurogenic regions, such as the SVZ [209,210]. Interestingly, and as the focus of this review, it has been reported that physical exercise can enhance hypothalamic neurogenesis [142]. In this article, Niwa et al. demonstrate that voluntary wheel running enhances neurogenesis and neuronal function in rodents. In this study, stroke-prone spontaneously hypertensive rats (SHRSP) and Wistar-Kyoto rats underwent voluntary aerobic exercise using running wheels, while the control groups remained sedentary. Starting at six weeks of age, the exercising rats ran freely on individual wheels, and their running distance was continuously recorded. This long-term experiment assessed survival rates, physiological parameters and histopathological outcomes, revealing the systemic effects of sustained voluntary aerobic activity compared to sedentary conditions. They found that exercise improved survival and energy balance in stroke-prone, hypertensive rats (SHRSP/Kpo), as well as promoting hypothalamic cell proliferation, particularly of tanycyte-like cells. This proliferation was found to correlate with increased FGF-2 expression in subependymal cells and CSF. Following a stroke, some of the newly formed cells matured into neurons, indicating that exercise-induced hypothalamic neurogenesis facilitates homeostatic recovery in the adult brain.

There is currently limited evidence regarding the direct effects of physical exercise on hypothalamic neurogenesis. However, several studies have found a potential link between the two, suggesting that physical exercise could be used as a therapeutic strategy for various diseases. Nishii and colleagues demonstrated that acute low-intensity treadmill training in rats increases the number of c-Fos-positive nuclei in the PVN and dorsal raphe nucleus of the hypothalamus, while also enhancing the number of DCX-positive cells in the hippocampus, which correlates with a reduction in depressive-like behavior [211]. The protocol used here was as follows: In this experiment, rats underwent a controlled aerobic treadmill exercise protocol. Following a 10-day habituation period involving a gradual increase in running speed (from 10 to 25 m/min) and duration (from 15 to 60 min), the animals were divided into three groups: sedentary controls, low-speed runners (15 m/min) and high-speed runners (25 m/min). The two exercising groups performed a single 30 min session of treadmill running during the dark phase of the light/dark cycle, while the control group remained on a stationary treadmill. The intervention aimed to evaluate the acute effects of low- and high-intensity aerobic exercise. While some studies suggest that hypothalamic neurogenesis may play a role in mediating the metabolic benefits of physical activity, other findings offer a contrasting perspective. Borg and his colleagues demonstrated the effects of physical exercise on hypothalamic neurogenesis and its potential to improve insulin sensitivity. The exercise protocol consisted of a short-term, moderate-intensity aerobic training regimen. The mice performed forced treadmill running at a speed of approximately 12 m per minute with a 5% incline for 30 min per day over seven consecutive days. This corresponds to a moderate-intensity continuous training (MICT) protocol that primarily engages oxidative metabolism. They found that short-term exercise induces a neurogenic transcriptional program in the hypothalamus and significantly increases cell proliferation, even in mice that are obese due to their diet. However, this proliferative response was not associated with neuronal differentiation within the ARC. Furthermore, inhibiting cell proliferation using arabinosylcytosine did not affect body mass, food intake or exercise-induced improvements in insulin sensitivity. Taken together, these findings suggest that exercise promotes significant non-neuronal cell proliferation in the hypothalamus, but that this process is not necessary for exercise to have a positive effect on insulin action [212]. Another study examined the impact of an HFD and physical exercise on neurogenesis and inflammation in the ARC of the hypothalamus in adult mice. This study demonstrates a model in which the combined effects of diet and exercise are examined. At six weeks of age, the mice were assigned to either a control diet or an HFD for twelve weeks. Within each dietary group, half of the animals were given access to a running wheel, enabling them to engage in spontaneous aerobic activity; the remaining animals remained sedentary. This voluntary wheel-running model represents sustained, low-to-moderate-intensity aerobic exercise that closely mimics natural physical activity in rodents. The results showed that an HFD increased both neurogenesis and microglial activation, whereas physical exercise stimulated cell proliferation and reduced the inflammatory response induced by an HFD. At a physiological level, exercise increased food and fat intake yet reduced the weight gain typically associated with an HFD. These results lend support to the hypothesis that hypothalamic neurogenesis acts as a compensatory mechanism in response to environmental or physiological stressors in order to preserve energy balance [156]. While not directly linked to neurogenesis, research has demonstrated that physical exercise can influence neural activity. For instance, aging has been found to decrease the density of GABAergic terminals in the PVN, which contributes to autonomic imbalance and cardiovascular dysfunction. Twelve weeks of aerobic exercise improved these parameters in aged rats, partially restoring GABAergic function and reducing the sympathetic overactivity associated with aging [213]. Other studies have revealed that the central dopaminergic system modulates physical performance via hypothalamic neuronal activation through D1 receptors. Experiments conducted on rats showed that blocking these receptors reduced the time taken to reach fatigue and increased c-Fos expression in thermoregulatory hypothalamic nuclei without affecting core body temperature. These results imply that dopamine plays a pivotal role in coordinating motor activity and hypothalamic activation during physical exercise [214]. As discussed, the most commonly used exercise programs are aerobics and include low-, moderate- and high-intensity protocols. But what about resistance exercise? There is also supporting evidence, although research in this area has primarily focused on other neurogenic niches, such as the hippocampus. Resistance exercise has also been shown to have a wide-ranging effect on neuroplasticity, with the distinctive feature of impacting muscle tissue simultaneously. The neuro-protective effects and role in promoting synaptic connectivity of several myokines have been studied [215]. As previously mentioned, resistance exercise is generally associated with the release of specific myokines, also known as exerkines, such as BDNF, IGF-1 and VEGF. These myokines mediate the exercise’s systemic and neurobiological effects. In fact, it has been shown that acute resistance exercise modulates BDNF levels, with a more pronounced response being elicited by higher intensities. This transient increase in BDNF following exercise may promote neuroplasticity, thereby enhancing learning and memory processes. However, the mechanisms by which resistance exercise links to BDNF regulation are unclear and require further investigation [216]. For example, Zuo et al. conducted a study examining the acute effects of resistance exercise on neurobiological factors in twelve young men. The participants performed high- and low-intensity training sessions (80% and 40% 1RM, respectively) in a randomized design. Both protocols increased lactate levels and reduced plasma homocysteine. However, high-intensity exercise induced greater elevations in BDNF, IGF-1 and VEGF. These results imply that resistance training, especially at a high intensity, can temporarily boost neurotrophic signaling and potentially provide neuroprotective benefits [217]. In an experimental animal study, Novaes Gomes and colleagues observed that resistance exercise enhanced hippocampal cell proliferation and modulated apoptotic signaling in rats. Specifically, four weeks of progressive resistance training increased the number of Ki67-positive cells in the DG, indicating elevated cell proliferation. However, these beneficial effects were negated when exercise was combined with the administration of nandrolone decanoate, which increased pro-apoptotic Bax immunoreactivity and reduced anti-apoptotic Bcl-2 expression. No significant changes in BDNF levels were detected across groups. These findings suggest that, although resistance training promotes hippocampal plasticity, exposure to anabolic steroids concurrently impairs these neuroprotective effects [218]. Conversely, Nokia et al. compared the effects of aerobic and resistance exercise on adult male rats. They discovered that voluntary running and treadmill endurance training significantly increased adult hippocampal neurogenesis. However, resistance training involving ladder climbing with weights, despite improving strength, had no effect on cell proliferation, maturation or the survival of newborn neurons. These results suggest that sustained aerobic exercise is more effective than resistance training in promoting adult hippocampal neurogenesis, especially in animals that are genetically predisposed to respond well to physical activity [219]. As previously mentioned, it is clear that a dedicated review would be required to address this broad yet inconsistent topic. This highlights the need for further, more comprehensive research in this area. In the case of the hypothalamus, the main focus of this review, the available evidence is diverse and somewhat limited. This is partly because resistance exercise influences the hypothalamus through complex mechanisms, primarily by modulating metabolic regulation and promoting neuroprotective factors such as IGF-1 and BDNF. While it is unclear whether these effects directly induce significant neurogenesis, they are known to enhance neuroplasticity and support neuronal function, particularly during moderate-intensity exercise [212,220]. The functional implications of hypothalamic neurogenesis remain largely unexplored, yet this area of research has great potential as a new way of tackling diseases involving neuronal loss and metabolic dysfunction. Different types of exercise may influence this process in different ways. For example, aerobic training of moderate and sustained intensity has been shown to enhance cell proliferation and trophic signaling in hypothalamic regions associated with energy balance and stress regulation. In contrast, resistance training appears to influence primarily systemic adaptations, such as increased circulating levels of IGF-1, BDNF, and anti-inflammatory cytokines, which may indirectly promote hypothalamic plasticity rather than robust neurogenesis. HIIT could offer the combined benefits of both the metabolic and neurotrophic effects, although evidence in hypothalamic contexts remains scarce. Overall, a better understanding of how different exercise programs modulate hypothalamic neurogenesis and neural remodeling could lead to new therapeutic approaches for neurodegenerative and metabolic disorders.

### 3.5. Nutraceuticals, Sports Supplements and Their Effects on the Neurogenic Niches

As previously defined, adult neurogenesis is modulated by intrinsic and extrinsic factors, including diet. While unhealthy habits suppress it, certain nutrients and lifestyle interventions, such as polyphenols, omega-3 fatty acids (PUFAs), caloric restriction and exercise, promote neurogenesis. Although the underlying mechanisms are unclear, nutrition offers a promising way to support brain function and counteract age-related cognitive decline and other neurodegenerative diseases [221,222]. It has been reported that several vitamins influence neurogenesis. One of the most significant is vitamin B9 (folate or folic acid), which plays a crucial role in nervous system development. Furthermore, a deficiency in folate has been linked to cognitive impairment, primarily due to elevated homocysteine levels, which increase the likelihood of developing Alzheimer’s disease. Furthermore, low folate levels have been shown to have a negative effect on hippocampal neurogenesis [223,224]. A deficiency in vitamin B12 has also been shown to impair cognitive function by either directly affecting hippocampal neurogenesis or disrupting axonal myelination [225]. Specifically in the hypothalamus, a deficiency in vitamin B9 and B12 can reduce the expression of the glucocorticoid receptor, which is a key component of the brain’s stress response system. However, the available evidence does not directly address the receptor’s role in hypothalamic neurogenesis [226].

In the case of vitamin E, deficiency has been linked to increased cell death in the DG of the hippocampus. This effect can be reversed through the use of α-tocopherol supplements [227]. Conversely, vitamin D deficiency has been reported to contribute to premature aging, reduced hippocampal neurogenesis and cognitive decline [228]. The effects of this deficiency on the hypothalamus are still being investigated, and we have not found any conclusive studies on neurogenesis in this area. Nevertheless, it is evident that it could indirectly impact hypothalamic function and must therefore be considered [229,230].

Research has primarily focused on PUFAs, which are essential for proper brain function. Several authors have reported that omega-3 has anti-inflammatory properties that are relevant to major depressive disorder and hippocampal neurogenesis [231,232]. Due to their neurogenic properties, PUFAs are considered promising dietary modulators that could promote neural plasticity and help maintain healthy hypothalamic function. In fact, most of the neurogenic activity induced by PUFAs was associated with an increased number of POMC neurons, but not NPY neurons. This was accompanied by elevated expression of BDNF and the G-protein-coupled receptor 40 (GPR40). Inhibiting GPR40 attenuated the neurogenic effects of PUFAs, whereas blocking BDNF reduced the overall number of hypothalamic cells. Therefore, PUFAs emerge as a promising dietary strategy for counteracting obesity-related neuronal loss in the hypothalamus [233].

Undoubtedly, phenolic compounds, which are considered nutraceuticals, represent one of the most promising areas of current research. These phytochemicals are derived from a wide variety of plants and are renowned for their extensive antioxidant and anti-inflammatory properties. Bioactive compounds found in berries, such as strawberries, as well as in grape seeds and skins, have been shown to have a positive effect on neurogenesis in the hippocampus and cognitive function, particularly memory [29,234,235,236]. Curcumin [237,238] and resveratrol [239,240] are among the most extensively studied phenolic compounds, having attracted considerable attention due to their neuroprotective, antioxidant and anti-inflammatory properties. In their comprehensive review, Ong et al. discuss the wide-ranging therapeutic potential of these compounds in the context of brain health and adult neurogenesis. However, they emphasize that further research is needed to identify their exact molecular targets and the mechanisms by which they act [241]. A study designed to evaluate the impact of resveratrol on hypothalamic neuronal dynamics in a diet-induced obesity model in mice provides an illustrative example of the effects of resveratrol on the hypothalamus. The study observed that HFD disrupts the balance of hypothalamic neurons by favoring orexigenic phenotypes, whereas resveratrol counteracts this effect by promoting the differentiation of anorexigenic POMC neurons. These results imply that resveratrol could potentially assist in the regulation of body weight and energy homeostasis by modulating hypothalamic neurogenesis [150]. Other studies have shown that resveratrol prevents age-related functional reprogramming of hypothalamic astrocytes in vitro. This reinforces its anti-aging properties and highlights its protective role within the hypothalamic environment [242]. There is limited information regarding the specific effects of curcumin on the hypothalamus. However, it is a promising compound for further investigation due to the positive results observed in other neurogenic niches. Its well-known antioxidant, anti-inflammatory and neuroprotective properties suggest it could play a part in preventing neuronal loss in hypothalamic structures, especially in the PVN. This nucleus is important for regulating stress responses and energy homeostasis [243].

When we focus specifically on the dietary supplements that athletes frequently use, creatine emerges as one of the most prominent, given its well-documented physiological and neurobiological effects. Leem et al. investigated the combined impact of regular exercise and creatine supplementation on hippocampal neurogenesis and depressive-like behavior in the context of chronic mild stress. Chronic stress was found to reduce neurogenesis in the DG and increase immobility in behavioral tests. The study showed that both interventions, particularly when combined, reversed these effects by activating the Wnt/GSK3β/β-catenin signaling pathway. Immunohistochemical and molecular analyses confirmed the restoration of neurogenic markers (Ki67 and doublecortin) and β-catenin nuclear translocation. Furthermore, local inhibition of Wnt signaling negated these benefits. Taken together, these findings suggest that creatine and exercise promote hippocampal neurogenesis and ameliorate depressive-like behaviors synergistically through activation of the Wnt pathway [244]. Furthermore, various studies have documented its positive impact on the prevention of neurodegenerative diseases [245,246,247,248,249]. A wide variety of supplements are commonly used by sport practitioners. However, it is important to emphasize that further preclinical studies are needed to determine their specific effects on adult neurogenesis. Nevertheless, understanding their mechanisms of action is crucial for identifying new compounds that could help to prevent cognitive decline associated with neuronal loss. In a narrative review, Antonio and colleagues analyzed the most evidence-based ergogenic aids used by physically active individuals. These were beta-alanine, nitrates, caffeine and protein. Beta-alanine increases levels of the muscle-buffering compound carnosine, delaying fatigue during short-duration, high-intensity exercise. Effective doses range from 2 to 6 g/day. Nitrates, primarily obtained from beetroot juice, enhance aerobic performance by improving oxygen delivery and reducing the body’s need for oxygen. Caffeine acts as a stimulant of the central nervous system, reducing perceived exertion and improving alertness and focus. Optimal doses are between 3 and 6 mg/kg. Protein supplementation supports muscle repair, growth and recovery, particularly after resistance training. Overall, these supplements demonstrate strong efficacy and safety profiles when used appropriately [250]. An overview of dietary supplements with potential benefits for brain health is presented in Table 3. As discussed previously, these compounds show great potential in the context of neurogenesis. However, further research is needed to fully understand their effects and the molecular mechanisms involved. To date, no study has determined the effect of such supplementation on hypothalamic neurogenesis in preclinical models, highlighting a significant gap in the literature and a promising area for future investigation.

## 4. Discussion

**Figure 2:**
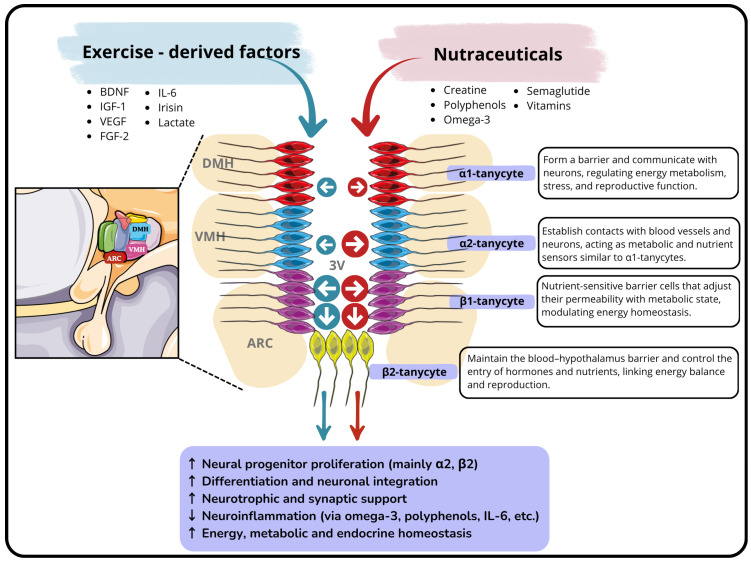
Convergent effects of exercise-derived factors and nutraceuticals on hypothalamic tanycytes and neurogenesis. On the left are the exercise-induced factors (BDNF, IGF-1, VEGF, FGF-2, IL-6, irisin and lactate), and on the right are the considered nutraceuticals (creatine, polyphenols, omega-3, semaglutide and vitamins). These signals reach the wall of the third ventricle (3V) and act on tanycyte subtypes (α1, α2, β1 and β2), thereby modulating barrier functions, metabolic sensing and communication with the DMH, VMH and ARC hypothalamic nuclei. The net outcomes are increased neural progenitor proliferation (mainly α2 and β2), increased neuronal differentiation and integration, increased neurotrophic and synaptic support, decreased neuroinflammation (e.g., via omega-3, polyphenols and IL-6) and improved energy, metabolic and endocrine homeostasis. Blue arrows denote exercise-derived influences and red arrows denote nutraceutical influences. Double arrows indicate the bidirectional exchange between the CSF, tanycytes and the hypothalamic parenchyma. Abbreviations: (ARC): arcuate nucleus; (DMH): Dorsomedial hypothalamic nucleus; (VMH): Ventromedial hypothalamic nucleus; (3V): third ventricle; (BDNF): brain-derived neurotrophic factor; (IGF-1): insulin-like growth factor 1; (VEGF): vascular endothelial growth factor; (FGF-2): fibroblast growth factor 2; (IL-6): interleukin 6; (ω-3/ω3): omega-3 polyunsaturated fatty acids. The arrows indicate: ↓ decreased; ↑ increased.

As discussed throughout this review, the hypothalamus is a relatively unexplored neurogenic niche among non-canonical regions. However, due to its potential role in regulating various biological processes, including energy balance, neuroendocrine function, stress responses and cognitive–affective behaviors, it is a neuroanatomical area of significant scientific interest. According to current definitions of a neurogenic niche and considering the specific cytoarchitecture that drives the activation or suppression of distinct signaling pathways influencing the capacity of a microenvironment to sustain NSCs, the hypothalamus can be regarded as a niche that is currently at the forefront of scientific research and investigation [134]. However, a deeper understanding of hypothalamic neurogenesis could therefore provide new insights into the plasticity of the adult brain and its role in health and disease. Conventional and unconventional neurogenic regions have been shown to exhibit distinct anatomical features, including the organization of cells within the brain parenchyma and their spatial relationship to the ventricles and, consequently, the CSF [302]. These anatomical particularities shape the cellular processes and dynamics within the niche, influencing the development of therapeutic strategies that may target these regions, as well as enabling the identification of potential pharmacological targets to counteract diseases associated with neuronal loss. While the majority of conclusive evidence has been obtained from animal models, whether neurogenesis persists in humans remains an active and unresolved area of investigation [119,303]. In several instances, the controversy has its origins in methodological limitations [304,305]. In the case of the hypothalamus, neurogenesis has been described in association with several of its nuclei; however, it is the proximity to the third ventricle that provides the signals required to sustain NSCs. It is evident that each of these nuclei is linked to key physiological functions (Table 1). Consequently, the modulation of hypothalamic neurogenesis could represent a significant step forward in the treatment of various disorders characterized by neuronal loss. The research focuses on the NSCs of the hypothalamus, of which the most prevalent are tanycytes [10,129]. These can be classified into four distinct subtypes: α1, α2, β1, and β2 [9,10,133]. The location of α1- and α2-tanycytes is primarily along the lateral walls of the third ventricle, and it is hypothesized that they participate in the regulation of hypothalamic neuronal activity through their interactions with adjacent neuronal populations. β1-tanycytes are located in closer proximity to the ARC, where they play a pivotal role in the regulation of energy balance and metabolic signaling. In contrast, β2-tanycytes are in direct contact with the ME and are essential for mediating exchanges between the CSF and the portal blood vessels, thereby establishing a link between neurogenesis and neuroendocrine regulation [21,306,307]. These tanycytes have been demonstrated to be amenable to in vitro cultivation, during which they manifest the capacity to form neurospheres [10]. In the course of research into adult neurogenesis, a range of compounds have been identified as modulators of this process, with effects on proliferation, differentiation, and neuronal survival. In recent years, there has been an increasing focus on plant-derived extracts and dietary supplements, with creatine in particular receiving attention. These compounds are not only frequently consumed within the sports community, including by amateur athletes, but also represent a growing area of interest due to their pleiotropic biological actions [247,308]. Beyond their recognized role in enhancing physical performance and energy metabolism, several of these substances have been shown to influence mechanisms of neuroplasticity, synaptic function, inflammation, and even adult neurogenesis in canonical brain regions such as the hippocampus and SVZ. Notwithstanding these advances, the potential impact on hypothalamic neurogenesis remains largely unexplored. Given the central role of the hypothalamus in energy homeostasis, neuroendocrine regulation, and behavior, elucidating the actions of these compounds on hypothalamic NSCs could open new avenues for therapeutic strategies targeting disorders associated with neuronal loss, metabolic dysregulation, or impaired neuroendocrine function [20]. A substantial body of evidence has emerged that underscores a robust correlation between adult neurogenesis, physical exercise, and dietary supplementation [213]. Evidence has repeatedly demonstrated that physical activity, specifically aerobic and high-intensity training, has a positive impact on neurogenesis [309]. This effect is achieved through various mechanisms, including enhanced cerebral blood flow, upregulation of neurotrophic factors such as BDNF and IGF-1, modulation of neurotransmitter systems, and attenuation of neuroinflammatory pathways [310,311]. Furthermore, several bioactive compounds and nutritional supplements have been shown to influence comparable molecular cascades, thereby supporting neuroplasticity, synaptic function, and neuronal survival. Examples of such compounds include creatine, polyphenols, omega-3 fatty acids, and certain vitamins [241]. As summarized in Figure 2, regular physical exercise, particularly aerobic training of a moderate to vigorous intensity, releases myokines and metabolites (e.g., BDNF, IGF-1, IL-6, irisin and lactate) that converge on hypothalamic tanycytes (α1–β2) along the ARC–VMH–DMH axis together with selected nutraceuticals. This process tunes barrier properties and metabolic sensing, thereby enhancing progenitor proliferation and neuronal differentiation while dampening neuroinflammation.

When considered together, exercise and supplementation may exert synergistic effects, promoting NSC proliferation, differentiation, and integration into functional networks. However, most of the extant evidence originates from studies conducted within canonical niches such as the hippocampus and the SVZ, with comparatively little attention being devoted to non-canonical regions such as the hypothalamus. Furthermore, the presence of variability in methodological approaches, dosage regimens, and the translational gap between animal models and humans persists as significant challenges. It is imperative to address these limitations to comprehensively understand the therapeutic potential of combining exercise and supplementation as modulators of adult neurogenesis, particularly in the context of disorders characterized by neuronal loss, metabolic imbalance, or impaired neuroendocrine regulation. In this review, we synthesize the available evidence to emphasize the urgent need for systematic investigations into hypothalamic neurogenesis and its modulation by physical exercise and dietary supplementation. While exercise and selected bioactive compounds (e.g., creatine, polyphenols, and omega-3 fatty acids) robustly influence neurogenic and neuroplasticity pathways in canonical niches (Table 3), their relevance to tanycyte-driven neurogenesis at the third ventricle remains insufficiently characterized. Key gaps are highlighted, including the scarcity of cell-type-resolved data, limited longitudinal and dose–response studies, and inconsistent outcome measures across preclinical and clinical research. These hinder causal inference and translational progress. In order to address these gaps, we propose integrative frameworks combining in vitro models of hypothalamic NSCs with in vivo paradigms of endurance and high-intensity training, coupled to multi-omics profiling, lineage tracing, and quantitative neuroendocrine readouts. The establishment of standardized protocols and clinically meaningful endpoints (metabolic control, neuroendocrine function, and cognitive–behavioral outcomes) is imperative to ascertain whether exercise and targeted supplementation act additively or synergistically on hypothalamic NSC proliferation, differentiation, and circuit integration. Furthermore, such an evaluation is necessary to determine their therapeutic potential in disorders characterized by neuronal loss, metabolic dysregulation, and impaired neuroendocrine regulation.

## 5. Conclusions

In this review, we outline the potential impact of various nutritional supplements on adult neurogenesis, paying particular attention to the hypothalamus, a non-canonical neurogenic niche which has not been widely studied. By integrating findings from nutritional neuroscience and exercise physiology, we highlight the importance of this region, not only for its central role in metabolic regulation, but also for its increasing significance in the pathophysiology of neuropsychiatric and neurodegenerative disorders. While most neurogenesis studies have focused on the hippocampus, there is a growing recognition that hypothalamic plasticity may play a comparable role in maintaining systemic and cognitive homeostasis. Therefore, there is an urgent need for targeted research to elucidate the influence of specific dietary components and ergogenic interventions on hypothalamic neural precursor populations and their microenvironment. As hypothalamic neurogenesis responds strongly to nutritional cues and physical activity, this field provides a promising basis for developing new therapeutic strategies to counteract neuronal loss, cognitive decline and metabolic dysfunction. Future research should adopt integrative, mechanistic and translational approaches capable of linking molecular pathways with functional outcomes, moving beyond descriptive studies. Specifically, (i) well-controlled preclinical studies are needed to characterize the responses of hypothalamic NSCs to defined nutritional and exercise stimuli, particularly compounds that are widely used in sports and clinical nutrition, such as PUFAs, resveratrol, curcumin, and creatine; (ii) secondly, given the regional and functional heterogeneity of the hypothalamus, it will be important to determine whether distinct nuclei, such as in the ARC, PVN, or VMH region, exhibit differential sensitivity to these interventions; (iii) the use of advanced molecular profiling techniques, including single-cell RNA sequencing, proteomics, and metabolomics, will be essential in identifying key regulatory networks, neurotrophic mediators, and biomarkers of neurogenic modulation. Translational and clinical studies are also required to bridge the gap between preclinical findings and human physiology by evaluating the combined effects of specific diets (e.g., the Mediterranean diet or a polyphenol-enriched diet) and structured exercise programs (e.g., aerobic exercise, HIIT, or resistance training) on metabolic, affective, and cognitive outcomes linked to hypothalamic function. Ultimately, targeting hypothalamic neurogenesis through nutritional and physical activity-based interventions represents a promising avenue to restore neural plasticity and energy homeostasis. This integrative perspective, bridging molecular neuroscience, physiology, and lifestyle medicine, may contribute to the prevention and treatment of both neurological and metabolic disorders, establishing the hypothalamus as a central node for future interdisciplinary research.

## Figures and Tables

**Table 1:** Functional and Signaling Features of Hypothalamic Nuclei.

Hypothalamic Nucleus	Main Function	Signaling Pathways
Tuberomammillary nucleus (TMN)	Control of **arousal**, **learning**, **memory** and **wakefulness** Functional inactivation produces **somnolence** [62,63]	Only source of **histamine** in brain; synthesized from Histidine by Histidine decarboxylase Acts on histamine receptors (H1–H4): **H1** → Neuronal depolarization → Promotes wakefulness **H2** → Modulates behavior **H3** → Inhibitory autoreceptor/heteroreceptor → Regulates multiple neurotransmitters **H4** → Mainly outside the central nervous system [64,65]
Lateral hypothalamic area (LHA)	Regulation of **feeding**, **drinking, energy balance**, **thermogenesis**, and **motivated behaviors** Lesions disrupt **feeding**, **drinking**, and **body weight regulation** Electrical stimulation promotes **ingestive behaviors** [66,67,68,69]	Distinct cell types exert opposing influences: **Orexin neurons:** ↑ food-seeking, arousal, and thermogenesis **Melanin-concentrating hormone (MCH) neurons:** ↑ feeding and fat storage and ↓ activity **Leptin receptor and neurotensin-expressing neurons:** ↓ hunger and ↑ exploration **Glucose-inhibited glutamic acid decarboxylase 65 (GAD65) neurons:** modulate feeding in response to energy status **Cells expressing solute carrier family 12 member 8 (Slc12a8)** influence systemic metabolism via sympathetic pathways [70,71,72]
Ventrolateral preoptic nucleus (VLPO)	Inhibiting subcortical arousal centers → **Promotion and maintenance of sleep** Lesions → **insomnia** Activation of γ-aminobutyric acid (GABA) → ↑ **wakefulness** [73,74]	Inhibit primarily **GABAergic** and **galaninergic neurons** projecting to orexin neurons in the LHA Some neurons co-express GABA and galanin Others express **excitatory markers** → **integrates inputs** from multiple brain regions → modulate arousal [73,75]
Arcuate nucleus (ARC)	Regulation of **feeding behavior**, **energy expenditure**, **glucose homeostasis**, **cardiovascular function**, and **fertility** [76,77]	Is richly endowed with **hormone receptors** and specialized **neuroendocrine cells** with distinct physiological roles: **Orexigenic agouti-related peptide** and **neuropeptide Y (NPY) neurons** → project to multiple hypothalamic targets → ↑ feeding and ↓ energy expenditure **Anorexigenic pro-opiomelanocortin** and **cocaine- and amphetamine-regulated transcript (CART) neurons** → release alpha-melanocyte-stimulating hormone (α-MSH) → ↓ appetite and ↑ metabolism These two populations function antagonistically. **Ghrelin** → excites orexigenic neurons and inhibits anorexigenic neurons **Leptin** and **insulin** → inhibit orexigenic neurons and activate anorexigenic neurons [77]
Ventromedial hypothalamic nucleus (VMH)	Satiety center of the central nervous system, playing a crucial role in regulating **food intake**, **glucose homeostasis**, **body weight** and **thermogenesis** Bilateral lesions → **overeating** and **obesity** Electrical stimulation of its neurons → ↓ **food consumption** [78,79,80]	Sends sympathetic and parasympathetic signals to visceral organs **AMP-activated protein kinase (AMPK) signaling**, **estrogen receptor alpha (ERα) activation**, and **melanocortin signaling** → Modulates brown and white adipose tissue Senses glucose through specialized neurons and regulates **glucagon** and **cortisol** [78,81]
Dorsomedial hypothalamic nucleus (DMH)	Plays a central role in regulating **feeding rhythms**, **food intake**, **brown adipose tissue thermogenesis**, **cardiorespiratory activity**, **neuroendocrine responses**, **stress avoidance**, **arousal**, **locomotor activity**, and **torpor** [82,83,84]	Specific DMH neurons sense energy balance and corticosterone levels, relaying this information to the LHA to coordinate adaptive responses. **Thyrotropin-releasing hormone (TRH) neurons** → project to various hypothalamic regions → depolarize orexin neurons → ↑ arousal and locomotor activity, and inhibit **MCH neurons** via local **GABAergic interneurons** **Leptin receptor-expressing neurons** → ↑ energy expenditure and ↓ body weight [82,84]
Suprachiasmatic nucleus (SCN)	Central pacemaker of the circadian timing system, regulating most **circadian rhythms** → **sleep–wake cycles**, **appetite**, **autonomic functions**, and **neuroendocrine activity** It modulates **hormone secretion** and **diurnal behaviors** based on light input received through the retinohypothalamic tract [85,86]	Most neurons are **GABA-positive** Core region enriched with **vasoactive intestinal polypeptide neurons** and shell region contains **arginine vasopressin (AVP) neurons** Receives **inhibitory GABAergic** input from the DMH → regulate food-anticipatory activity Regulates **glucocorticoid** rhythms through projections to the PVN, modulating **corticotropin-releasing hormone (CRH)** release Connects to the ARC to influence feeding and energy balance through **α-MSH neurons** [87]
Anterior hypothalamic nucleus (AHN)	Modulation of **heat loss**, **metabolic heat**, **defensive attacks**, **social aggression**, and **thirst/fluid balance** [88,89,90,91]	**Vasopressin-expressing neurons (via V1a receptor) →** stress responses, social behavior **Noradrenergic activity →** thirst regulation, fluid homeostasis, autonomic adjustments [92,93]
Paraventricular nucleus (PVN)	Control **arousal**, **defensive behavior**, **pain perception**, the **hypothalamic–pituitary–adrenal axis**, **hypothalamic–pituitary–thyroid axis**, **growth hormone axis**, **hydromineral balance**, **uterine contraction**, **milk letdown**, **appetite**, and **metabolism** Dysfunctions **→ obesity**, **insomnia**, **anxiety**, **depression**, and **chronic pain** [94,95]	Diverse neuronal populations producing distinct hormones: **CRH, TRH, oxytocin, AVP, glutamate/neuropeptides** Beyond direct endocrine control, sends **glutamatergic** and **neuropeptidergic** projections to multiple brain regions [94,96]
Posterior hypothalamic nucleus (PHN)	Regulates **heat production**, **sympathetic activity**, **heart rate**, **blood pressure**, **alertness**, and **defensive behaviors** Local inhibition decreases **body temperature** and **heart rate** under cold stress [97,98,99,100]	Receives **afferents** from the insular cortex, septal nuclei, amygdala, subiculum, bed nucleus of stria terminalis, central gray, parabrachial nucleus, nucleus of the solitary tract and brainstem reticular nuclei Contains a mix of **glutamate-releasing**, **GABA-releasing**, **peptide-producing** (such as **orexin**, **MCH**, and **NPY**), and **catecholamine-releasing neurons** Expresses a wide range of receptors including **GABA-A**, **N-methyl-D-aspartate (NMDA)**, **alpha-amino-3-hydroxy-5-methyl-4-isoxazolepropionic acid (AMPA)**, **orexin**, **corticotropin-releasing factor**, **muscarinic acetylcholine**, **adrenergic**, and **dopamine** receptors [97,101,102,103,104,105]
Supraoptic nucleus (SON)	Play essential roles in **water homeostasis**, **lactation**, and **reproductive behaviors** [106,107]	Composed of **magnocellular neuroendocrine cells** (MNCs) **→** including **vasopressin-** and **oxytocin-producing neurons →** regulate **vasopressin** secretion Receives **projections** from the MPO, ARC, and parabrachial nuclei [106,107]
Medial preoptic nucleus (MPO)	Play a central role in **sexual behavior, parental care**, **aggression**, **social interactions and social behaviors** [108,109,110]	Integrates **hormonal signals**: **oxytocin**, **prolactin**, **estrogen**, and **progesterone** Relies on specific neuronal subtypes: **galanin-**, **neurotensin-**, and **GABA-expressing neurons** **Hormone-sensitive neurons** convert endocrine signals into activity patterns → **project** to ventral tegmental area and periaqueductal gray Spatial organization defines hormonal integration [58,108]
Median preoptic nucleus (MnPO)	Contributes to **body fluid balance**, integrates **thermal** and **immune signals**, mediating **fever** and regulate **sleep–wake states** [111,112,113]	Expresses **angiotensin type 1** receptors, allowing **angiotensin II** to increase neuronal excitability → regulate thirst, blood pressure, and AVP release via **MnPO → PVN/SON projections** **Prostaglandin E_2_** acts on **prostaglandin E_2_ receptor subtype 3-expressing (EP3R) neurons** → mediate fever **Vesicular glutamate transporter 2 (Vglut2) glutamatergic neurons** → wakefulness **Vesicular GABA transporter (Vgat) GABAergic neurons** → non-rapid eye movement sleep [111,112,113]

Functional and signaling characteristics of the major hypothalamic nuclei. This table summarizes the main functions of each nucleus and the signaling pathways associated with them. Abbreviations: (TMN): tuberomammillary nucleus; (H1–H4): histamine receptors types 1–4; (LHA): lateral hypothalamic area; (MCH): melanin-concentrating hormone; (GAD65): glutamic acid decarboxylase 65; (Slc12a8): solute carrier family 12 member 8; (VLPO): ventrolateral preoptic nucleus; (GABA): γ-aminobutyric acid; (ARC): arcuate nucleus; (NPY): neuropeptide Y; (CART): cocaine- and amphetamine-regulated transcript; (α-MSH): alpha-melanocyte-stimulating hormone; (VMH): ventromedial hypothalamic nucleus; (AMPK): AMP-activated protein kinase; (Erα): estrogen receptor alpha; (DMH): dorsomedial hypothalamic nucleus; (TRH): thyrotropin-releasing hormone; (SCN): suprachiasmatic nucleus; (AVP): arginine vasopressin; (CRH): corticotropin-releasing hormone; (AHN): anterior hypothalamic nucleus; (PVN): paraventricular nucleus; (SON): supraoptic nucleus; (MNCs): magnocellular neuroendocrine cells; (MPO): medial preoptic nucleus; (PHN): posterior hypothalamic nucleus; (NMDA): N-methyl-D-aspartate; (AMPA): alpha-amino-3-hydroxy-5-methyl-4-isoxazolepropionic acid; (MnPO): median preoptic nucleus; (EP3R): prostaglandin E_2_ receptor subtype 3; (Vglut2): vesicular glutamate transporter 2; (Vgat): vesicular GABA transporter. The arrows indicate:↓ decreased; → effect or consequence; ↑ increased.

**Table 2:** Studies Characterizing Adult Neurogenesis in Hypothalamic Nuclei.

Hypothalamic Nucleus	Author and Year (Ref)	Animal Species	Neurogenic Markers	Treatment	Conclusion
Lateral hypothalamic area (LHA)	Chaker et al., 2016 [137]	CAG-tdTomato/0 mice **♀**	**BrdU^+^/NeuN^+^ Tom^+^/NeuN^+^**	Tamoxifen induction of Cre recombinase	Although the LHA is functionally important, to our knowledge, **evidence for adult neurogenesis in this region is limited** to a single study reporting widespread neurogenesis across multiple hypothalamic nuclei, including the LHA.
Arcuate nucleus (ARC)	Safahani et al., 2018 [150]	C57BL/6 mice **♂**	**BrdU^+^/POMC^+^ BrdU^+^/NPY^+^**	High-fat diet (HFD) exposure	Although only the most relevant articles are highlighted here, **evidence for adult neurogenesis in the ARC is extensive** and it seems to be mainly modulated by dietary factors such as an HFD and exercise.
	Lee et al., 2014 [151]	C57BL/6 mice **♀/♂**	**BrdU^+^/Hu^+^**	HFD exposure	
	Bless et al., 2014 [152]	C57BL6 mice **♀**	**BrdU^+^/Hu^+^**	HFD exposure	
	Gouazé et al., 2013 [153]	C57BL/6 mice **♂**	**BrdU** ^+^ **/NeuN** ^+^ **BrdU** ^+^ **/POMC** ^+^	HFD exposure	
	Chaker et al., 2016 [137]	CAG-tdTomato/0 mice **♀**	**BrdU^+^/NeuN^+^ Tom^+^/NeuN^+^**	Tamoxifen induction of Cre recombinase	
	Batailler et al., 2014 [154]	C57BL/6 mice **♀/♂**	**Nestin^+^/Sox2^+^/DCX^+^**	None	
	Jörgensen et al., 2023 [155]	C57BL/6 mice **♂**	**BrdU^+^/NeuN^+^**	HFD exposure, Prolactin-Releasing Peptide administration, and Liraglutide administration	
	Klein et al., 2019 [156]	C57BL/6 mice **♀**	**BrdU** ^+^ **/HuD** ^+^ **BrdU** ^+^ **/POMC** ^+^	HFD and exercise exposure	
Ventromedial hypothalamic nucleus (VMH)	Bless et al., 2014 [152]	C57BL/6 mice **♀**	**BrdU^+^/Hu^+^**	HFD exposure	Similar to the ARC, **extensive studies have demonstrated adult neurogenesis in the VMH**, which appears to be modulated by dietary and hormonal factors. Certain subpopulations of newborn neurons have been identified as responsive to hormones such as estrogens and leptin, indicating functional integration into metabolic circuits.
	Chaker et al., 2016 [137]	CAG-tdTomato/0 mice **♀**	**BrdU^+^/NeuN^+^ Tom^+^/NeuN^+^**	Tamoxifen induction of Cre recombinase	
	Batailler et al., 2014 [154]	C57BL/6 mice **♀/♂**	**Nestin^+^/Sox2^+^/DCX^+^**	None	
	Feighan et al., 2024 [157]	C57BL/6 mice **♀/♂**	**BrdU^+^/NeuN^+^**	Gestational Bisphenol A (BPA) and HFD offspring exposure	
	Levy et al., 2019 [158]	Ile de France ewes **♀**	**Ki67^+^/DCX^+^** **Sox2^+^/DCX^+^**	Steroid-primed and oxytocin infusion	
	Jörgensen et al., 2023 [155]	C57BL/6 mice **♂**	**BrdU^+^/NeuN^+^**	HFD exposure, Prolactin-Releasing Peptide administration, and Liraglutide administration	
Dorsomedial hypothalamic nucleus (DMH)	Bless et al., 2014 [152]	C57BL/6 mice **♀**	**BrdU^+^/Hu^+^**	HFD exposure	While less extensively studied than the ARC or VMH, **evidence for adult neurogenesis in the DMH has been reported.**
	Chaker et al., 2016 [137]	CAG-tdTomato/0 mice **♀**	**BrdU^+^/NeuN^+^ Tom^+^/NeuN^+^**	Tamoxifen induction of Cre recombinase	
	Feighan et al., 2024 [157]	C57BL/6 mice **♀/♂**	**BrdU^+^/NeuN^+^**	Gestational Bisphenol A (BPA) and HFD offspring exposure	
	Jörgensen et al., 2023 [155]	C57BL/6 mice **♂**	**BrdU^+^/NeuN^+^**	HFD exposure, Prolactin-Releasing Peptide administration, and Liraglutide administration	
Paraventricular nucleus (PVN)	Feighan et al., 2024 [157]	C57BL/6 mice **♀/♂**	**BrdU^+^/NeuN^+^**	Gestational Bisphenol A (BPA) and HFD offspring exposure	**Evidence for adult neurogenesis in the PVN is limited** but consistent across species. New neurons can be generated under various physiological and environmental conditions, such as gestational BPA exposure, HFD, and WD.
	Raymond et al., 2006 [159]	Yorkshire pigs **♀**	**PCNA^+^/OT^+^**	None	
	Zhang et al., 2024 [160]	Wistar and Sprague–Dawley rats **♀/♂**	**BrdU^+^/NPII^+^** **DCX^+^/NPII^+^**	Chronic intermittent water-deprivation (WD)	
Posterior hypothalamic nucleus (PHN)	Chaker et al., 2016 [137]	CAG-tdTomato/0 mice **♀**	**BrdU^+^/NeuN^+^ Tom^+^/NeuN^+^**	Tamoxifen induction of Cre recombinase	To our knowledge, evidence for **adult neurogenesis in the PHN is limited**, and no studies have specifically investigated this nucleus.
Preoptic area of anterior hypothalamus (POA/AH)	Raymond et al., 2006 [159]	Yorkshire pigs **♀**	**PCNA^+^/OT^+^**	None	While the specific nuclei within the PO/AH have not been individually investigated for adult neurogenesis, **evidence consistently supports the occurrence of this process in the PO/AH** as a whole, modulated by factors such as heat exposure and WD.
	Matsuzaki et al., 2015 [161]	Wistar rats **♂**	**BrdU^+^/NeuN^+^**	Heat exposure	
	Shido and Matsuzaki, 2015 [162]	Wistar rats **♂**	**BrdU^+^/NeuN^+^**	Heat exposure	
	Matsuzaki et al., 2017 [141]	Wistar rats **♂**	**BrdU^+^/NeuN^+^**	Heat exposure	
	Zhang et al., 2024 [160]	Wistar and Sprague–Dawley rats **♀/♂**	**BrdU^+^/NPII^+^** **DCX^+^/NPII^+^**	WD	

Studies Characterizing Adult Neurogenesis in Hypothalamic Nuclei. This table summarizes the current evidence regarding adult neurogenesis in hypothalamic nuclei. Abbreviations: (LHA): lateral hypothalamic area; (BrdU): 5-bromo-2′-deoxyuridine; (NeuN): neuronal nuclei marker RBFOX3; (Cre): Cre recombinase; (ARC): arcuate nucleus; (POMC): pro-opiomelanocortin; (NPY): neuropeptide Y; (HFD): high-fat diet; (Hu): pan-Hu neuronal antigen; (Nestin): neural progenitor intermediate filament; (Sox2): SRY-box transcription factor 2; (DCX): doublecortin; (VMH): ventromedial hypothalamic nucleus; (BPA): bisphenol A; (DMH): dorsomedial hypothalamic nucleus; (PVN): paraventricular nucleus; (PCNA): proliferating cell nuclear antigen; (NPII): neurophysin II; (PHN): posterior hypothalamic nucleus; (POA/AH): preoptic area/anterior hypothalamus; (WD): water-deprivation.

**Table 3:** Effects of Dietary Supplement Compounds on Adult Neurogenesis.

Compound	Proposed Mechanism of Action	Effects on Exercise Performance	Author and Year (ref)	Dose and Duration	Neurogenic Evidence
**Caffeine**	Adenosine receptor antagonism, **↑ endorphin** release [251]	**↑ Performance →** **↑ Muscle endurance** **↑ Strength** **↑ Anaerobic power** **↑ Aerobic capacity** [251,252]	Tiwari et al., 2023 [253]	10 mg/kg intra-peritoneally (i.p.), once daily for 28 days	**↑ BrdU^+^/DCX^+^** **↑ BrdU^+^/NeuN^+^** Niche: Hippocampus
			Stazi et al., 2021 [254]	300 mg/L in drinking water for 4 months	**↓ Neuron loss** **↑ DCX^+^** Niche: Hippocampus
			Han et al., 2007 [255]	0.3 g/L in drinking water for 4 weeks	**↓ Proliferation of neural stem cells (NSCs)** Niche: Hippocampus
			Wentz et al., 2009 [256]	20–60 mg/kg per day in saline (0.02 mL/g body weight) for 7 days	Intermediate doses: **↓ BrdU^+^** highest dose: **↑ BrdU^+^** **↔ Differentiation** and **survival** Niche: Hippocampus
			Mao et al., 2020 [257]	10–20 mg/kg, oral (intragastric) daily for 4 weeks	**↑ BrdU^+^/DCX^+^** Niche: Hippocampus
			Stazi et al., 2023 [258]	300 mg/L in drinking water, chronic administration for 4 months	**↔ DCX^+^ cells** Niche: Hippocampus
			Endesfelder et al., 2018 [259]	10 mg/kg, i.p., daily for 3 consecutive days	↑ **Ki67^+^/NeuN^+^** **↑ DCX^+^** Niche: Hippocampus
			Houghton et al., 2020 [260]	0.1–1.0 mM	Low dose: **↔ DAPI^+^, Nestin^+^, SOX2^+^** High dose: **↓ DAPI^+^, Nestin^+^, SOX2^+^** **↔Ki67^+^/CC3^+^** In vitro
**Creatine**	**↑ Brain creatine stores** → **↑ phosphocreatine resynthesis** [248,251]	**↑** **Lean body mass** **↑ Muscle strength** **↑ Performance** in short, high-intensity, repetitive activities **Anti-inflammatory** and **antioxidant benefits** [251,261]	Leem et al., 2018 [244]	Oral via food, 4% of pellet, 4 weeks	**↑Ki-67^+^/DCX^+^** Niche: Hippocampus
			Yang et al., 2025 [262]	100 μM	**↑ HuC/D**^+^ **↑ neurite length** In vitro
			Pazini et al., 2017 [263]	10 mg/kg, oral gavage, once daily for 21 days	**↑Ki-67^+^** **↑NeuroD^+^** versus corticosterone Niche: Hippocampus
**Nitrate**	**↑ nitric oxide** → **↑ AMPK**, **glucose uptake**, **insulin** and **endothelial function**. **↓ oxidative stress** and **fat synthesis** [251,264]	**↑ Endurance performance** → **↑ Muscle oxygenation ↑ Mitochondrial efficiency** **↑ Contractile function**. **↑ Type II muscle fiber function** [251,265]	Vercalsteren et al., 2025 [264]	0.1 mmol/kg/day, oral via drinking water, 18 weeks	**↔ Ki67^+^** **↔ DCX^+^** Niche: subventricular zone (SVZ)
			Zhang et al., 2001 [266]	0.1–0.4 mg/kg/day, intravenous or i.p., for 7 days	**↑ BrdU^+^ cells** Niche: Hippocampus, SVZ
**Curcumin**	**Anti-inflammatory,** **↓ oxidative stress** [251,267]	**↑ Recovery** **↓ Inflammation** **↓ Oxidative stress** **↑ Cardiovascular response** **↑ Thermoregulatory response** **↑ Psychological response** [267]	Dong et al., 2012 [268]	Oral via chow, 480 mg/kg for 6–12 weeks	**↑ BrdU^+^** Niche: Hippocampus
			Kim et al., 2008 [269]	0.1, 0.5, 1, 10, 20, and 50 μm 500 nmol/kg, i.p., once daily for 4 days	**↑ BrdU^+^** **↑ BrdU^+^/NeuN^+^** Niche: Hippocampus, In vitro
			Li et al., 2025 [270]	10–20 mg/mL in 0.9% saline, administered by gavage for 7 days	**↑ BrdU^+^** **↑ dendritic growth.** Niche: Hippocampus
			Lee et al., 2023 [271]	Oral, once daily at 0.4, 2, or 10 mg/kg for 14 days	**↑ BrdU^+^** **↑ BrdU^+^/NeuN^+^** **↑ DCX^+^** [251,267] Niche: Hippocampus
			Yang et al., 2021 [272]	50, 100 mg/kg/day i.p.	**↑ Brdu^+^** **↑ DCX^+^** **↑ Brdu^+^/NeuN^+^** Niche: Hippocampus.
			Lou et al., 2024 [237]	100 mg/kg, intragastric, 14 days	**↑ BrdU^+^** **↑ BrdU^+^/DCX^+^** Niche: Hippocampus.
			Chen et al., 2025 [273]	0 µM, 0.5 µM, 2.5 µM, 12.5 µM, 62.5 µM 100 mg/kg and 300 mg/kg	**↑ Proliferation of NSCs** **↑ EdU^+^/NeuN^+^** Niche: Hippocampus, In vitro
**Melatonin**	**Regulating circadian rhythms** and the **sleep–wake cycle.** **Neuroprotective**, **antioxidant**, **anti-inflammatory**, and **anti-apoptotic** effects [274]	**↓ Oxidative stress** **↓ Inflammation** **↓ Muscle damage** **↓ Liver damage** **↑ Recovery** of **muscle function** [275]	Li et al., 2017 [276]	100 nM for 1, 3, 5, or 7 days	**↓ Nestin^+^/DAPI^+^** **↑ Tuj1^+^** **↑ MAP2^+^** **↔ GFAP** In vitro
			Ghareghani et al., 2017 [277]	0.05, 0.1, 0.5, 1, 5 and 10 μM	Low dose: **↑ Viability of NSCs** **↑ MBP^+^** **↑ GFAP^+^** In vitro
			Liu et al., 2016 [278]	10 μM	**↑ PC12 cell proliferation** **↑ Neurite outgrowth** **↑ MAP2^+^** In vitro
			Sharma et al., 2008 [279]	1.0 and 10 nM	**↑ Neurite-like extensions** **↑ mRNA expression of Nestin** **↑ mRNA expression of** **β-III-tubulin** In vitro
			Ramirez-Rodriguez et al., 2011 [280]	8 mg/kg/day, i.p. for 14 days	**↑ DCX^+^** **↑ Dendritic maturation** Niche: Hippocampus
			Rennie et al., 2009 [281]	0.51 mg/kg/day, oral (drinking water), for 7 days	**↑ DCX^+^** **↑ BrdU^+^/NeuN^+^** Niche: Hippocampus
			Motta-Teixeira et al., 2018 [282]	0.5 mg/kg/day, oral (drinking water), for 7 days	**↑ Ki-67^+^** versus maternal melatonin deprivation Niche: Hippocampus
			Ramírez-Rodríguez et al., 2012 [283]	8 mg/kg/day, oral, for 3, 6, 9, or 12 months	**↑ Ki67^+^** **↑ pH3H^+^** **↑ BrdU**^+^ **↑ DCX**^+^ Niche: Hippocampus
			Liu et al., 2013 [284]	0.02 mg/mL/day, oral for 12 days	**↑ BrdU^+^/NeuN^+^** Niche: Hippocampus
			Ortiz-López et al., 2016 [285]	Chronic blockade of melatonin membrane receptors	Chronic blockade of melatonin membrane receptors: **↓ Ki67^+^** **↓ DCX^+^** Niche: Hippocampus
			Vega-Rivera et al., 2020 [286]	2.5 mg/kg/day, i.p., for 4 weeks	**↑ Ki-67^+^** **↑ BrdU^+^** **↑ DCX^+^** **↑ Dendritic complexity** versus chronic mild stress Niche: Hippocampus
**Resveratrol**	**Protective** effects against **vascular** and **neurodegenerative diseases**, **atherosclerosis**, **oxidative damage**, and **some cancers.** **Antioxidant activity** [274]	**↓ Delayed onset muscle soreness** **↓ Muscle damage** **↓ Inflammation** **↓ Oxidative stress** **↑ Recovery** [287]	Dasgupta et al., 2007 [288]	10 μM	**↑ Proliferation** **↑ neurite outgrowth** In vitro
			Thomas et al., 2014 [289]	50 mg/kg/day mixed with diet for 6 weeks	**↑ Neurogenesis** **↑ synaptic plasticity** **↑ Hdac4, Hat1, Wnt7a, ApoE** versus diabetic control Niche: Hippocampus
			Kodali et al., 2015 [290]	40 mg/kg/day dissolved in 0.5 mL of 2% ethyl alcohol for 4 weeks	**↑ BrdU**^+^ **↑ DCX**^+^ **↔NeuN^+^** Niche: Hippocampus
			Park et al., 2012 [291]	0.1, 1, 10, 20, 50 μM 1 or 10 mg/kg/day, i.p., for 14 days	*Higher doses:* In vitro: **↓ Proliferation of NSCs** **↓ Survival of NSCs** In vivo: **↓ BrdU^+^** **↓ DCX^+^** **↓ NeuN^+^/BrdU^+^** Niche: Hippocampus, In vitro
**omega-3 fatty acids** (**PUFAs)**	Amongst PUFAs: **α-linolenic acid, eicosapentaenoic acid** (EPA) **Docosahexaenoic acid** (DHA) → **Anti-oxidative stress**, **anti-inflammatory** and **antiapoptotic effects** [292]	**↑ Muscle recovery** **↓ Inflammation** **↓ Oxidative stress** **↓ Muscle damage** **↑ Muscle protein synthesis** [251,293]	Borsini et al., 2020 [232]	10 µM for 7 days	EPA and DHA: **↑ DCX^+^** DHA: **↑ Map2^+^** EPA: **↓ CC3+ apoptosis** Against IFN-α, EPA and DHA: **↑ DCX^+^** **↑ Map2^+^** In vitro
			Beltz et al., 2007 [294]	27.2 mg/g oral 25 days	**↑ BrdU^+^** Niche: Hippocampus
			He et al., 2009 [295]	In vitro: 5 µM DHA In vivo: DHA by diet for 10–12 weeks	DHA, in vitro: **↑ Tuj1^+^** **↑ BrdU^+^** **↑ Neurite length** DHA, in vivo: **↑ BrdU^+^** Niche: Hippocampus, In vitro
			Kawakita et al., 2006 [296]	DHA 10 µM for 4–7 days	DHA, in vitro: **↑ Tuj1^+^** **↓ BrdU^+^** **↑ Neurite length** DHA, in vivo: **↑ BrdU+/NeuN+** Niche: Hippocampus, In vitro
			Rodríguez-Iglesias et al., 2022 [297]	Ω6/Ω3 ratio 6.7 for 10 weeks	**↑ Nestin^+^** **↑ GFAP^+^** **↑ DCX^+^** **↑ BrdU^+^/DCX^+^** **↑ DCX^+^ dendritic extension** Niche: Hippocampus
			Dyall et al., 2010 [298]	270 mg/kg/day, EPA to DHA ratio (1.5:1) for 12 weeks	**↑ DCX^+^** versus age-related decline Niche: Hippocampus.
			Huguet et al., 2023 [299]	2 g DHA/100 g fat for 10 weeks	**↑ DCX^+^** Niche: Hippocampus, Hyphotalamus
**β-alanine**	**Anti-inflammatory**, **antioxidant**, **antiglycation**, **anticarbonylation**, **calcium-regulatory**, **immunomodulatory**, and **chelating properties** [251,300]	**↑ Performance** for continuous and intermittent exercise [251]	Gibbons et al., 2014 [301]	417 mg/kg/day for 28 days	**↔ BrdU** **↔ gene expression** Niche: Hippocampus

This table summarizes bioactive compounds for which there is evidence of effects on proliferation or differentiation in distinct neurogenic niches. It highlights their potential relevance in sports supplementation and exercise-related applications. Abbreviations: (BrdU): 5-bromo-2′-deoxyuridine; (DCX): doublecortin; (NeuN): neuronal nuclei marker RBFOX3; (i.p.): intraperitoneal; (DAPI): 4′,6-diamidino-2-phenylindole; (NSCs): neural stem cells; (SVZ): subventricular zone; (Nestin): neural progenitor intermediate filament; (SOX2): SRY-box transcription factor 2; (CC3): cleaved caspase-3; (Hu): neuronal RNA-binding proteins; (AMPK): AMP-activated protein kinase; (EdU): 5-ethynyl-2′-deoxyuridine; (Tuj1): class III β-tubulin; (MAP2): microtubule-associated protein 2; (GFAP): glial fibrillary acidic protein; (MBP): myelin basic protein; (pH3): phospho-histone H3; (Hdac4): histone deacetylase 4; (Hat1): histone acetyltransferase 1; (Wnt7a): Wnt family member 7A; (ApoE): apolipoprotein E; (EPA): eicosapentaenoic acid; (DHA): docosahexaenoic acid; (PC12): pheochromocytoma cell line. The arrows indicate: ↓ decreased; ↔ no change; ↑ increased.

## Data Availability

No new data were created or analyzed in this study. Data sharing is not applicable to this article.

## Abbreviations

The following abbreviations are used in this manuscript:

(ACTH): adrenocorticotropic hormone; (ADH): antidiuretic hormone; (AHN): anterior hypothalamic nucleus; (AMPA): alpha-amino-3-hydroxy-5-methyl-4-isoxazolepropionic acid; (AMPK): AMP-activated protein kinase; (ApoE): apolipoprotein E; (ARC): arcuate nucleus; (AVP): arginine vasopressin; (BDNF): brain-derived neurotrophic factor; (BPA): bisphenol A; (BrdU): 5-bromo-2′-deoxyuridine; (CART): cocaine- and amphetamine-regulated transcript; (CC3): cleaved caspase-3; (CRH): corticotropin-releasing hormone; (CSF): cerebrospinal fluid; (DAPI): 4′,6-diamidino-2-phenylindole; (DCX): doublecortin; (DG): dentate gyrus; (DHA): docosahexaenoic acid; (DMH): dorsomedial hypothalamic nucleus; (DMAR): dorsomedial Arcuate region; (DMN): dorsomedial nucleus of the hypothalamus; (EdU): 5-ethynyl-2′-deoxyuridine; (EPA): eicosapentaenoic acid; (EP3R): prostaglandin E_2_ receptor subtype 3; (ERα): estrogen receptor alpha; (FGF): fibroblast growth factor; (FGF-2): fibroblast growth factor 2; (FGF10): fibroblast growth factor 10; (GABA): γ-aminobutyric acid; (GAD65): glutamic acid decarboxylase 65; (GFAP): glial fibrillary acidic protein; (GLAST): Glutamate Aspartate Transporter; (GPR40): G-protein-coupled receptor 40; (GR): glucocorticoid receptors; (H1–H4): histamine receptors types 1–4; (Hat1): histone acetyltransferase 1; (Hdac4): histone deacetylase 4; (HFD): high-fat diet; (HIIT): high-intensity interval training; (HPA): Hypothalamic–Pituitary–Adrenal axis; (HSR): high-speed runners; (Hu): pan-Hu neuronal antigen; (IGF-1): insulin-like growth factor-1; (IL): interleukin; (IL-6): interleukin 6; (i.p.): intraperitoneal; (LHA): lateral hypothalamic area; (MAP2): microtubule-associated protein 2; (MBP): myelin basic protein; (MCH): melanin-concentrating hormone; (ME): median eminence; (MeSH): Medical Subject Headings; (MICT): moderate-intensity continuous training; (MnPO): median preoptic nucleus; (MNCs): magnocellular neuroendocrine cells; (MPO): medial preoptic nucleus; (MPTP): 1-methyl-4-phenyl-1,2,3,6-tetrahydropyridine; (mTOR): mechanistic target of rapamycin; (mTORC1): mTOR complex 1; (Nestin): neural progenitor intermediate filament; (NeuN): neuronal nuclei marker RBFOX3; (NLRP3): NLR family pyrin domain containing 3; (NMDA): N-methyl-D-aspartate; (NPII): neurophysin II; (NPY): neuropeptide Y; (NPY/AgRP): Neuropeptide Y/agouti-related peptide; (NSCs): neural stem cells; (OB): olfactory bulb; (OVLT): vascular organ of the lamina terminalis; (PC12): pheochromocytoma cell line; (PCNA): proliferating cell nuclear antigen; (PHN): posterior hypothalamic nucleus; (pH3): phospho-histone H3; (POA): preoptic area; (POA/AH): preoptic area / anterior hypothalamus; (POMC): pro-opiomelanocortin; (POMC/CART): Pro-opiomelanocortin/cocaine- and amphetamine-regulated transcript; (PUFAs): omega-3 fatty acids; (PVN): paraventricular nucleus; (SCN): suprachiasmatic nucleus; (SHRSP): stroke-prone spontaneously hypertensive rats; (SHRSP/Kpo): hypertensive rats; (SIRT1): sirtuin 1; (Slc12a8): solute carrier family 12 member 8; (SON): supraoptic nucleus; (Sox2/SOX2): SRY-box transcription factor 2; (SVZ): subventricular zone; (TMN): tuberomammillary nucleus; (TRH): thyrotropin-releasing hormone; (Tuj1): class III β-tubulin; (UCP1): uncoupling protein 1; (VEGF): vascular endothelial growth factor; (Vgat): vesicular GABA transporter; (Vglut2): vesicular glutamate transporter 2; (VLPO): ventrolateral preoptic nucleus; (VMARH): ventromedial Arcuate nucleus of the Hypothalamus; (VMH): ventromedial hypothalamic nucleus; (VMN): ventromedial nucleus of the hypothalamus; (WD): water-deprivation; (Wnt7a): Wnt family member 7A; (ω-3/ω3): omega-3 polyunsaturated fatty acids.
